# A novel hypoxic long noncoding RNA KB-1980E6.3 maintains breast cancer stem cell stemness via interacting with IGF2BP1 to facilitate c-Myc mRNA stability

**DOI:** 10.1038/s41388-020-01638-9

**Published:** 2021-01-19

**Authors:** Pengpeng Zhu, Fang He, Yixuan Hou, Gang Tu, Qiao Li, Ting Jin, Huan Zeng, Yilu Qin, Xueying Wan, Yina Qiao, Yuxiang Qiu, Yong Teng, Manran Liu

**Affiliations:** 1 Key Laboratory of Laboratory Medical Diagnostics, Chinese Ministry of Education, Chongqing Medical University, Chongqing, 400016 China; 2 Department of pharmacy, The First Affiliated Hospital of Chongqing Medical University, Chongqing, 400016 China; 3 Experimental Teaching Center of Basic Medicine Science, Chongqing Medical University, Chongqing, 400016 China; 4 Department of Endocrine and Breast Surgery, The First Affiliated Hospital of Chongqing Medical University, Chongqing, 400016 China; 5 Department of Oral Biology and Dx Sciences, Dental College of Georgia; Georgia Cancer Center, Augusta University, Augusta, GA 30907 USA

**Keywords:** Breast cancer, Non-coding RNAs, Cancer stem cells

## Abstract

The hostile hypoxic microenvironment takes primary responsibility for the rapid expansion of breast cancer tumors. However, the underlying mechanism is not fully understood. Here, using RNA sequencing (RNA-seq) analysis, we identified a hypoxia-induced long noncoding RNA (lncRNA) KB-1980E6.3, which is aberrantly upregulated in clinical breast cancer tissues and closely correlated with poor prognosis of breast cancer patients. The enhanced lncRNA KB-1980E6.3 facilitates breast cancer stem cells (BCSCs) self-renewal and tumorigenesis under hypoxic microenvironment both in vitro and in vivo. Mechanistically, lncRNA KB-1980E6.3 recruited insulin-like growth factor 2 mRNA-binding protein 1 (IGF2BP1) to form a lncRNA KB-1980E6.3/IGF2BP1/c-Myc signaling axis that retained the stability of c-Myc mRNA through increasing binding of IGF2BP1 with m6A-modified c-Myc coding region instability determinant (CRD) mRNA. In conclusion, we confirm that lncRNA KB-1980E6.3 maintains the stemness of BCSCs through lncRNA KB-1980E6.3/IGF2BP1/c-Myc axis and suggest that disrupting this axis might provide a new therapeutic target for refractory hypoxic tumors.

## Introduction

Hypoxia fractions in tumor microenvironments, caused by insufficient vascularization and high tumor metabolic and proliferation rates, contribute to the rapid development of many solid tumors including breast cancer [1–3]. Despite recent advances in screening and diagnostic tools, breast cancer remains the most common malignancy and the second leading cause of cancer-related mortality for women worldwide [4, 5]. The high rates of tumor incidence and death associated with this cancer are linked to diverse factors that are underscored by its intratumoral heterogeneous nature [6]. As a subpopulation of tumor cells, breast cancer stem cells (BCSCs) display a strong self-renewal ability and multidirectional differentiation potential. Conventional cancer therapies, therefore, are insufficient for eradicating BCSCs due to their highly resistant nature, leading to poorer therapeutic results [7–9]. Consequently, it is of particular importance to unveil the detailed regulatory mechanism of hypoxia in breast cancer from the perspective of BCSCs, which may aid in implementing personalized treatment strategies for breast carcinomas.

Long noncoding RNAs (lncRNAs) are a series of transcript RNAs greater than 200 nucleotides that have limited or no protein-coding capacity. Increasing evidence demonstrates the pivotal role of lncRNAs in governing a plethora of cancer-related cellular processes, such as proliferation, invasion, migration, apoptosis, and stemness [10–12]. LncRNAs may serve as oncogenic regulators through various mechanisms, including chromatin modification, genomic imprinting, and transcriptional, and posttranscriptional regulation, and thus, may contribute to cancer formation and progression [13–16].

Interestingly, a specific group of lncRNAs are modulated by tumor microenvironmental conditions, such as hypoxia. These hypoxia-responsive lncRNAs (HRLs), such as NORAD, LncHIFCAR, RAB11B-AS1, and AC020978, may underlie the survival of cancer cells and promote disease progression [17–20]. HRLs can be categorized into two subgroups, hypoxia-inducible factor (HIF)-dependent and HIF-independent, with the former subgroup composing the majority of HRLs. In hypoxic tumor microenvironments, HIF can directly bind with the hypoxia response element (HRE) located in the promoter regions of HRLs and regulate its expression. The abnormally expressed HRLs could activate some tumor-specific molecular profile and further contribute to tumor hallmarks [21–24]. Despite this, the biological characteristics of HRLs in breast cancer remain elusive.

A vast array of m6A RNA readers, such as YT521-B homology (YTH) domain-containing proteins (YTHDF1, YTHDF2, YTHDF3, YTHDC1, YTHDC2) and insulin-like growth factor 2 mRNA-binding proteins (IGF2BP1, IGF2BP2, IGF2BP3) can recognize m6A-modified RNA and control its fate by affecting mRNA stability, translation, alternative splicing, and subcellular localization [25–27]. Among them, IGF2BP1 has the potential to regulate gene stability through recognizing m6A-modified RNA of target genes, which further facilitates cancer progression [25, 28].

In this context, we performed RNA sequencing (RNA-seq) profiling to define HRLs in breast cancer. The KB-1980E6.3 (also named AP002852.1) is located at human chromosome 8q22.3 and gives rise to two transcripts, KB-1980E6.3-001 (Transcript Accession ENST00000523572.1) and KB-1980E6.3-002 (Transcript Accession ENST00000519630.1). After filtration, we identified KB-1980E6.3-001 as a hypoxia-induced target, which locates in chr8:102528755-102529801. LncRNA KB-1980E6.3 was significantly upregulated in breast tumor tissues compared with normal tissues and closely associated with short survival of breast cancer patients. Functional research revealed that lncRNA KB-1980E6.3 significantly promotes BCSCs stem-like properties by binding to IGF2BP1 to enhance the stability of c-Myc mRNA under hypoxia conditions. Collectively, our data demonstrate that lncRNA KB-1980E6.3 plays a critical role in breast cancer progression and might act as a potential therapeutic target.

## Results

### LncRNA KB-1980E6.3 is a new hypoxic lncRNA and correlated with a poor prognosis in breast cancer

**Fig. 1:**
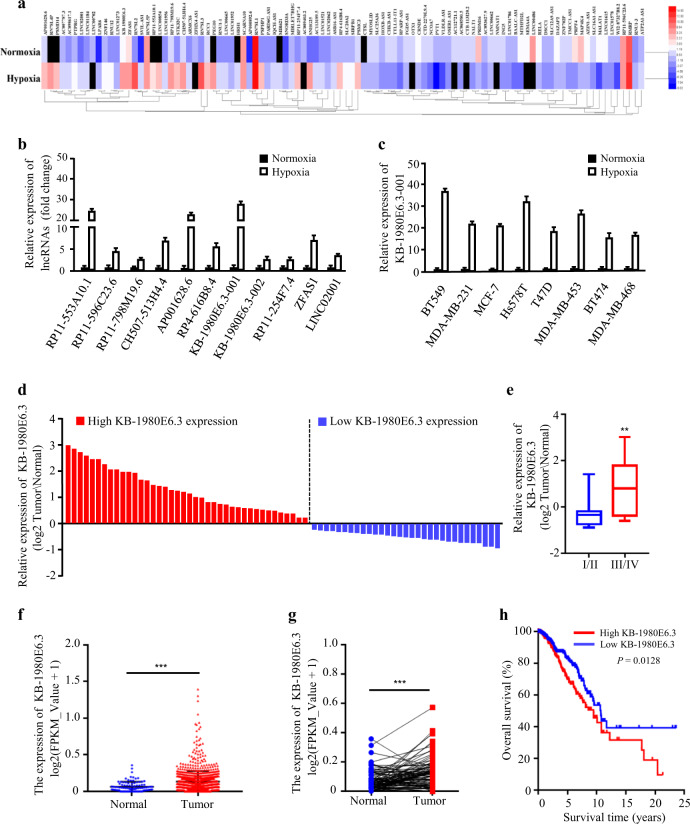
LncRNA KB-1980E6.3 is upregulated by hypoxia and correlated with a poor prognosis in breast cancer. **a** The heat map from RNA sequencing analysis showing the top 100 differentially expressed lncRNAs in hypoxic BT549 cells compared with normoxic BT549 cells. **b** Expression of 11 randomly selected upregulated lncRNAs were verified by qRT-PCR in hypoxic BT549 cells compared with normoxic BT549 cells. **c** qRT-PCR was performed to determine the lncRNA KB-1980E6.3 expression in various hypoxic breast cancer cells compared with that in normoxic breast cancer cells. **d** The heights of the columns in the chart represent the log2-transformed fold changes (tumor vs normal) in lncRNA KB-1980E6.3 expression in 71 paired breast cancer tissues and adjacent non-cancerous tissues. **e** The relative log2-transformed fold changes (tumor vs normal) of lncRNA KB-1980E6.3 in 71 cases of breast tumors with different clinical stages were measured by qRT-PCR. **f**, **g** The lncRNA KB-1980E6.3 expression levels in non-paired (**f**) or paired (**g**) breast tumor tissues and normal tissues based on the RNA-seq data extracted from the TCGA database. **h** Kaplan–Meier survival analysis of overall survival according to the lncRNA KB-1980E6.3 expression levels based on TCGA cohort. Data are shown as mean ± SD of three independent experiments (**P < 0.01; ***P < 0.001).

Hypoxia is a common feature in solid tumors and plays key roles in tumor development. To explore the effects of hypoxia on breast tumor malignancy, we performed an RNA-seq analysis to acquire genomic expression profiles of BT549 cells exposed to normoxia (20% O_2_) or hypoxia (1% O_2_) for 8 h. One hundred eighty-one upregulated and 229 downregulated lncRNAs were identified under hypoxia conditions (Fig. S1a), and the top 100 differentially expressed lncRNAs were depicted by heat map (Fig. 1a). To validate the RNA-seq analysis data, we randomly chose 11 upregulated lncRNAs with a fold change greater than five times (*p* < 0.01) to test their expression levels in BT549 cells cultured at normoxia or hypoxia for 8 h by quantitative real-time PCR (qRT-PCR). Of which, lncRNA KB-1980E6.3-001 (Transcript Accession ENST00000523572.1) was the most increased lncRNA under hypoxia conditions when compared with normoxia (Fig. 1b). Moreover, the upregulated lncRNA KB-1980E6.3-001 was further verified in other hypoxic breast cancer cells, especially in hypoxic BT549 and Hs578T cells (Fig. 1c); thus, these two cells were chosen for subsequent functional studies. To further confirm its hypoxia-dependence in breast cancer cells, BT549 and Hs578T cells were cultured under hypoxia conditions at varying time intervals (0, 6, 12, 24, and 48 h) and a gradual increase in lncRNA KB-1980E6.3 expression was detected (Fig. S1b). We then analyzed the correlation between lncRNA KB-1980E6.3 and the hypoxia-responsive genes in breast cancer tissues from The Cancer Genome Atlas (TCGA) and found that lncRNA KB-1980E6.3 was positively correlated with VEGFA (*r* = 0.4593, *P* < 0.001), SLC2A1 (*r* = 0.4182, *P* < 0.001), P4HA1 (*r* = 0.4341, *P* < 0.001), and CA9 (*r* = 0.5421, *P* < 0.001) (Fig. S1c), the known targets of HIF in breast cancer.

The sequences of lncRNAKB-1980E6.3 have been listed in Supplementary Table 1. The online prediction software Coding Potential Calculator (CPC) (http://cpc.cbi.pku.edu.cn/) and RNA coding potential assessment tool (CPAT) (http://lilab.research.bcm.edu/cpat/index.php) were performed to predict the potential protein-coding capacity of lncRNA KB-1980E6.3 and the data showed that lncRNA KB-1980E6.3 was a noncoding RNA (Fig. S1d). After analyzing its nuclear and cytoplasmic RNA fractionation, we revealed that lncRNA KB-1980E6.3 was mainly located in the cytoplasm of hypoxic BT549 and Hs578T cells (Fig. S1e).

We next evaluated the expression level of lncRNA KB-1980E6.3 in 71 pairs of breast tumors and their adjacent normal tissues and found that most tumor tissues (40/71) showed higher levels of lncRNA KB-1980E6.3 when compared with adjacent normal tissues (Fig. 1d). Moreover, increased lncRNA KB-1980E6.3 was positively related to tumor stage of breast cancer patients (Fig. 1e and Table 1). We then conducted an RNA-seq data analysis including 1100 breast cancer specimens and 113 normal specimens from TCGA database to expand our findings. Consistent with our aforementioned data, higher expression of lncRNA KB-1980E6.3 was found in breast cancer tissues compared with adjacent non-cancerous tissues (Fig. 1f, g). Kaplan–Meier survival curve demonstrated that a higher level of lncRNA KB-1980E6.3 was closely correlated with poor survival of breast cancer patients (Fig. 1h). These results highlight the clinical significance of lncRNA KB-1980E6.3 in breast cancer.

**Table 1:** Correlation between KB-1980E6.3 expression and clinicopathologic characteristics of breast cancer patients.

Characteristics	All cases	KB-1980E6.3		*P* value
		Low	High	
All cases	71	31	40	
Age				
<60	33	18	15	0.085
≥60	38	13	25	
T				
T1	29	23	6	<0.001*
T2/3/4	42	8	34	
N				
N0/1	39	21	18	0.056
N2/3	32	10	22	
M				
M0	35	19	16	0.075
M1	36	12	24	
Stage				
I/II	23	18	5	<0.001*
III/IV	48	13	35	

*P < 0.05.

### LncRNA KB-1980E6.3 is upregulated by HIF-1α during hypoxia

**Fig. 2:**
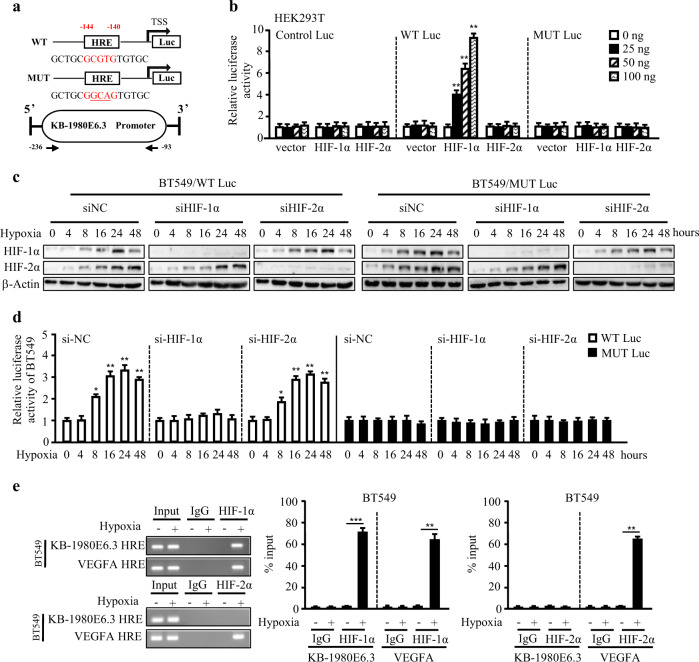
HIF-1α binds to the HRE in lncRNA KB-1980E6.3 promoter to regulate lncRNA KB-1980E6.3 transcription. **a** Schematic illustration of the putative HRE in the promoter of lncRNA KB-1980E6.3, the WT and MUT of lncRNA KB-1980E6.3 luciferase promoter vector construct. “**→**” arrow indicates the location of primers used in ChIP assay. **b** HEK293T cells were transfected with pGL3-lncRNA KB-1980E6.3 WT reporter, pGL3-lncRNA KB-1980E6.3 MUT reporter or the control reporter combined with HIF-1α or HIF-2α (0, 25, 50, and 100 ng) encoding vectors and cultured under normoxia conditions for 24 h. The Dual-luciferase reporter activity was analyzed. **c** BT549 cells were transfected with pGL3-lncRNA KB-1980E6.3 WT reporter, pGL3-lncRNA KB-1980E6.3 MUT reporter combined with siHIF-1α or siHIF-2α under hypoxia conditions. The expression levels of endogenous HIF-1α or HIF-2α at designed time point (0, 4, 8, 16, 24, and 48 h) were checked by western blotting. **d** BT549 cells were transfected with pGL3-lncRNA KB-1980E6.3 WT reporter, pGL3-lncRNA KB-1980E6.3 MUT reporter coupling with siHIF-1α or siHIF-2α under hypoxia conditions. Dual-luciferase reporter activity was tested at the designed hypoxia exposure time (0, 4, 8, 16, 24, and 48 h). **e** ChIP assay used to measure the binding of HIF-1α or HIF-2α on the lncRNA KB-1980E6.3 promoter in BT549 cells under hypoxia conditions. The binding on the VEGFA promoter served as a positive control for the HIF response. Data are shown as mean ± SD of three independent experiments (*P < 0.05; **P < 0.01; ***P < 0.001).

HIF is the key hypoxia-dependent transcriptional factor in regulating hypoxia-associated gene expression and promoting the malignant process of breast cancer. To confirm whether HIF promotes the expression of lncRNA KB-1980E6.3 in breast cancer cells, we used lentivirus-mediated shRNAs against HIF-1α or HIF-2α to knockdown HIF expression in BT549 and Hs578T cells (Fig. S2a–d), which was further confirmed by expression of VEGFA, a known HIF target under hypoxia conditions (Fig. S2e, f). Indeed, hypoxic lncRNA KB-1980E6.3 was significantly decreased in HIF-1α knockdown breast cancer cells rather than in HIF-2α silenced cells under hypoxia conditions. Further, low levels of lncRNA KB-1980E6.3 were detected in normoxic HIF-1α or HIF-2α silenced cells, indicating that lncRNA KB-1980E6.3 is an HIF-1α dependent lncRNA under hypoxia conditions (Fig. S2g, h). HIF-1α or HIF-2α plasmids were additionally transfected into BT549 and Hs578T cells and it was found that the transient transfection of HIF-1α, rather than HIF-2α, increased lncRNA KB-1980E6.3 levels in BT549 and Hs578T cells under normoxic conditions (Fig. S2i). From analysis using bio-informatics, we identified an HRE (−144 to −140 bp) in the promoter of lncRNA KB-1980E6.3 (Fig. 2a). To further evaluate HIF-1α’s involvement in the regulation of lncRNA KB-1980E6.3 expression, luciferase reporter assay and chromatin immunoprecipitation (ChIP) assay were conducted. Transfection of pGL3-lncRNA KB-1980E6.3 wild-type (WT) reporter combined with HIF-1α or HIF-2α plasmid in HEK293T cells showed HIF-1α, rather than HIF-2α, increased lncRNA KB-1980E6.3 HRE-driven luciferase activity in a dose-dependent manner in normoxic HEK293T cells (Fig. 2b, middle panel). However, HRE mutation in the promoter of lncRNA KB-1980E6.3 abolished its transcript activity caused by HIF-1α (Fig. 2b, right panel). To further verify these findings, we co-transfected pGL3-lncRNA KB-1980E6.3 WT reporter combined with siHIF-1α or siHIF-2α into hypoxic BT549 and Hs578T cells, and found that the loss of HIF-1α, rather than HIF-2α (Figs. 2c and S3a), significantly decreased lncRNA KB-1980E6.3 HRE-driven luciferase activity in BT549 and Hs578T cells (Figs. 2d and S3b, left panels). In addition, the HRE mutation in lncRNA KB-1980E6.3 promoter impaired the hypoxia-induced luciferase activities in BT549 and Hs578T cells (Figs. 2d and S3b, right panels). Consistent with the luciferase data, ChIP assay also validated that HIF-1α, rather than HIF-2α, could bind to the HRE site of lncRNA KB-1980E6.3 promoter in hypoxic BT549 or Hs578T cells (Figs. 2e and S3c). These data demonstrate that hypoxia-stimulated HIF-1α, but not HIF-2α, plays a role in regulating lncRNA KB-1980E6.3 expression.

### Hypoxia-mediated enhanced lncRNA KB-1980E6.3 promotes stemness maintenance of BCSCs

**Fig. 3:**
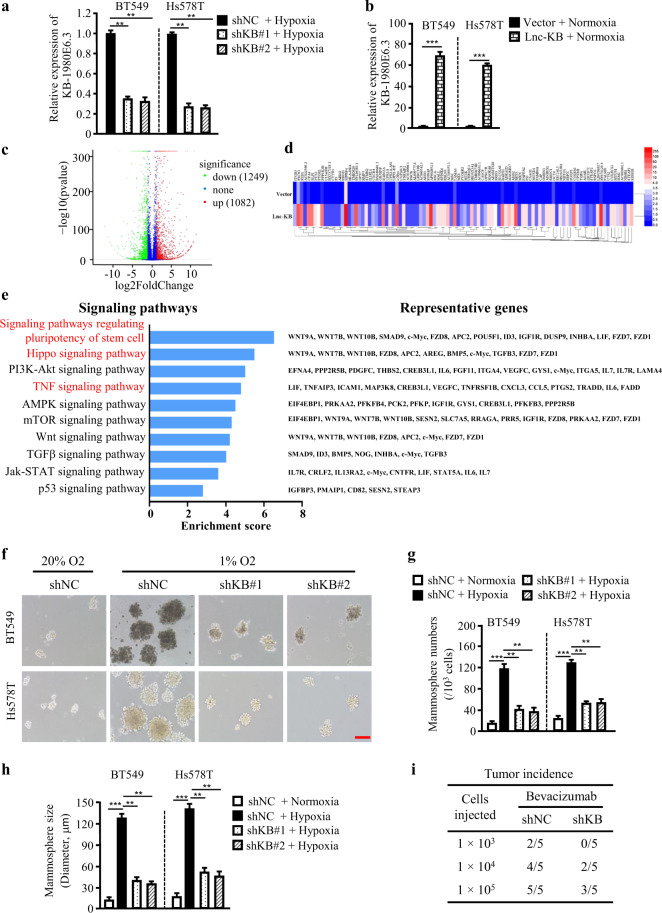
LncRNA KB-1980E6.3 functions to retain characteristics of breast cancer stem cell. **a**, **b** Knockdown or overexpression efficiencies of lncRNA KB-1980E6.3 were assessed by qRT-PCR analysis. **c** The volcano plot showing the differential genes between lncRNA KB-1980E6.3 overexpressing tumor cells and their control cells under normoxia conditions. **d** The heat map showing the top 100 upregulated genes between lncRNA KB-1980E6.3 overexpressing tumor cells and their control cells under normoxia conditions. **e** KEGG pathway analysis of differentially expressed genes in (**c**). Representative genes were shown in the right panel. **f**–**h** Mammosphere formation abilities were assessed in lncRNA KB-1980E6.3 knockdown and control breast cancer cells. Representative images of spheres, mammosphere numbers, and mammosphere sizes are shown in (**f**), (**g**), and (**h**), respectively. Scale bar, 100 μm. **i** Tumor initiation incidence in nude mice injected with indicated sphere cells from Hs578T shNC or shKB under administration of bevacizumab (10 mg/kg). Data are shown as mean ± SD of three independent experiments (**P < 0.01; ***P < 0.001).

To explore the function of lncRNA KB-1980E6.3 in breast cancer cells, lncRNA KB-1980E6.3 stable knockdown or overexpressed BT549 and Hs578T cells were established and utilized as cell models (Fig. 3a, b). RNA-seq analysis showed that 2331 genes (1082 upregulated and 1249 downregulated) were differentially expressed in the lncRNA KB-1980E6.3 overexpressed tumor cells in comparison to the control cells under normoxic conditions (Fig. 3c, d), and 537 genes (324 upregulated and 213 downregulated) were differentially expressed in the lncRNA KB-1980E6.3 knockdown tumor cells in comparison to the control cells under hypoxia conditions (Fig. S3d, e). The genes were then classified by using Kyoto Encyclopedia of Genes and Genomes (KEGG) pathway database (Figs. 3e and S3f). Interestingly, TNF signaling pathway, Hippo signaling pathway, and signaling pathways regulating pluripotency of stem cells were identified in both lncRNA KB-1980E6.3 knockdown and overexpressing cell models. Signaling pathways regulating the pluripotency of stem cells, which are associated with disease recurrence and metastasis, caught our attention among these signaling pathways, suggesting that lncRNA KB-1980E6.3 may play a role in BCSCs characteristics. Indeed, hypoxia could notably stimulate spheroid formation of breast cancer cells in suspended culture and loss of lncRNA KB-1980E6.3 significantly abrogated the spheroid formation abilities of breast cancer cells under hypoxia conditions (Fig. 3f–h), whereas ectopic lncRNA KB-1980E6.3 overexpressing breast cancer cells acquired strong spheroid formation potentials in comparison with their controls under normoxia conditions (Fig. S4a–c). Consistently, clone survival of breast cancer cells was strikingly increased under hypoxia conditions, while the loss of lncRNA KB-1980E6.3 reduced clone survival; however, ectopic lncRNA KB-1980E6.3 notably increased clone survival of breast cancer cells under normoxic conditions (Fig. S4d–g).

CSCs are essential for tumor initiation. To understand whether lncRNA KB-1980E6.3 mediated changes in BCSCs characteristics that impact tumorigenicity, we tested breast tumor initiation using mammosphere cells from Hs578T in vivo. Three doses (1 × 10^5^, 1 × 10^4^, and 1 × 10^3^) of spheres derived from lncRNA KB-1980E6.3 silenced Hs578T cells (labeled as shKB) or control cells (labeled as shNC) were subcutaneously injected into 4- to 6-week-old female nude mice (*n* = 5 per group), respectively. These mice were also treated with bevacizumab to form hypoxic microenvironment in tumor. As shown in Fig. 3i and Fig. S4h, at least 1 × 10^4^ spheres derived from shKB were required to generate tumors in xenograft mice, whereas 1 × 10^3^ spheres derived from shNC could form tumors in xenograft mice. LncRNA KB-1980E6.3 knockdown attenuated tumor growth and tumor weight of xenograft mice (Fig. S4i, j). These data suggest that lncRNA KB-1980E6.3 is essential for the maintenance of BCSCs stemness and tumorigenic ability of breast cancer cells.

### LncRNA KB-1980E6.3 enhances c-Myc protein levels by increasing c-Myc mRNA stability

**Fig. 4:**
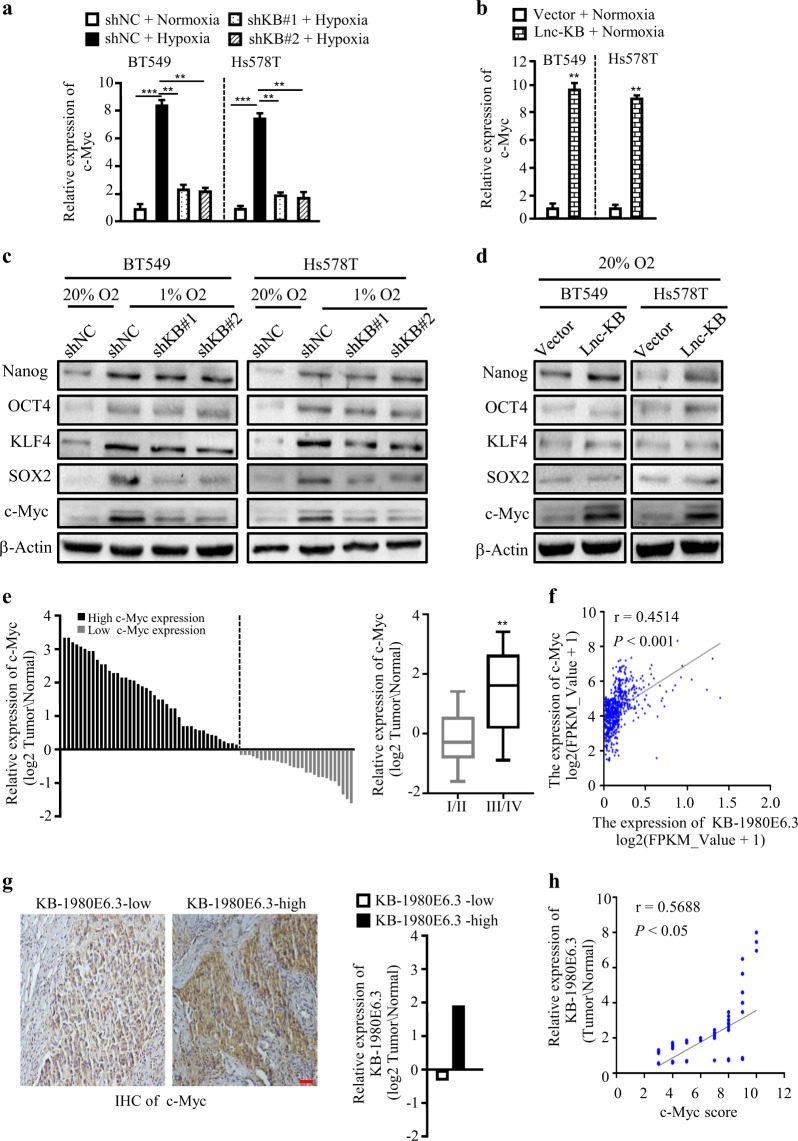
LncRNA KB-1980E6.3 is positive correlated with c-Myc expression. **a**, **b** c-Myc mRNA levels in lncRNA KB-1980E6.3 knockdown BT549 and Hs578T cells under hypoxia conditions (**a**) or lncRNA KB-1980E6.3 overexpressing BT549 and Hs578T cells under normoxia conditions (**b**). **c**, **d** The protein levels of c-Myc, KLF4, SOX2, OCT4, and Nanog in lncRNA KB-1980E6.3 knockdown BT549 and Hs578T cells under hypoxia conditions (**c**) or lncRNA KB-1980E6.3 overexpressing BT549 and Hs578T cells under normoxia conditions (**d**). **e** The heights of the columns in the chart represent the log2-transformed fold changes (tumor vs normal) in c-Myc expression in 71 paired breast cancer tissues and adjacent non-cancerous tissues (left panel). The relative log2-transformed fold changes (tumor vs normal) of c-Myc in 71 cases of breast tumors with different clinical stages were measured by qRT-PCR (right panel). **f** The correlation between c-Myc mRNA and lncRNA KB-1980E6.3 expression levels in breast cancer patients based on the data from TCGA database. **g** Representative IHC staining images showing c-Myc protein levels in lncRNA KB-1980E6.3 high or low tumor tissues (left panel). Scale bar, 50 μm. LncRNA KB-1980E6.3 levels in the lncRNA KB-1980E6.3 high or low tumor groups (right panel). **h** The correlation between lncRNA KB-1980E6.3 RNA level and c-Myc protein IHC scores. Data are shown as mean ± SD of three independent experiments (**P < 0.01; ***P < 0.001).

It is well known that c-Myc, KLF4, SOX2, OCT4, and Nanog are the major regulators involved in BCSCs stemness and self-renewal functions. Thus, we asked whether lncRNA KB-1980E6.3 might contribute to BCSCs formation by regulating some of these CSC-associated gene or protein expressions. Confirmed by qRT-PCR and western blot, we found that lncRNA KB-1980E6.3 knockdown had less impact on CSC-associated gene expression in normoxic conditions (Fig. S5a, b). However, lncRNA KB-1980E6.3 knockdown decreased the expression levels of CSC-associated genes (Fig. S6a), especially the c-Myc gene in hypoxic BT549 and Hs578T cells (Fig. 4a); ectopic lncRNA KB-1980E6.3 could increase these gene expressions (Fig. S6b), particularly the c-Myc gene in normoxic BT549 and Hs578T cells (Fig. 4b). Similarly, effect of lncRNA KB-1980E6.3 on c-Myc protein level was more apparent than other pluripotency-associated markers (e.g. OCT4, KLF4, SOX2, and Nanog), which western blotting further validated (Fig. 4c, d), suggesting that hypoxic lncRNA KB-1980E6.3 is closely correlated with c-Myc expression.

To expand our findings, we assessed HIF-1α, c-Myc, and CD44 levels in our clinic breast tumor tissues. Coinciding with lncRNA KB-1980E6.3, higher levels of HIF-1α, c-Myc, and CD44 were detected in most tumor tissues, and their levels were increased in accompany with tumor stages (Fig. S6c, d and Fig. 4e). After analyzing the correlation of c-Myc and lncRNA KB-1980E6.3 levels in breast cancer tissues from TCGA, we further found a positive correlation between c-Myc mRNA and lncRNA KB-1980E6.3 levels (*r* = 0.4514, *P* < 0.001) in breast tumors (Fig. 4f). More importantly, in our clinic breast tumor tissues, we found that c-Myc protein were much higher in lncRNA KB-1980E6.3-high tumor tissues than those in lncRNA KB-1980E6.3-low tumor tissues (Fig. 4g), and there was a positive correlation between lncRNA KB-1980E6.3 levels and c-Myc protein scores (Fig. 4h).

**Fig. 5:**
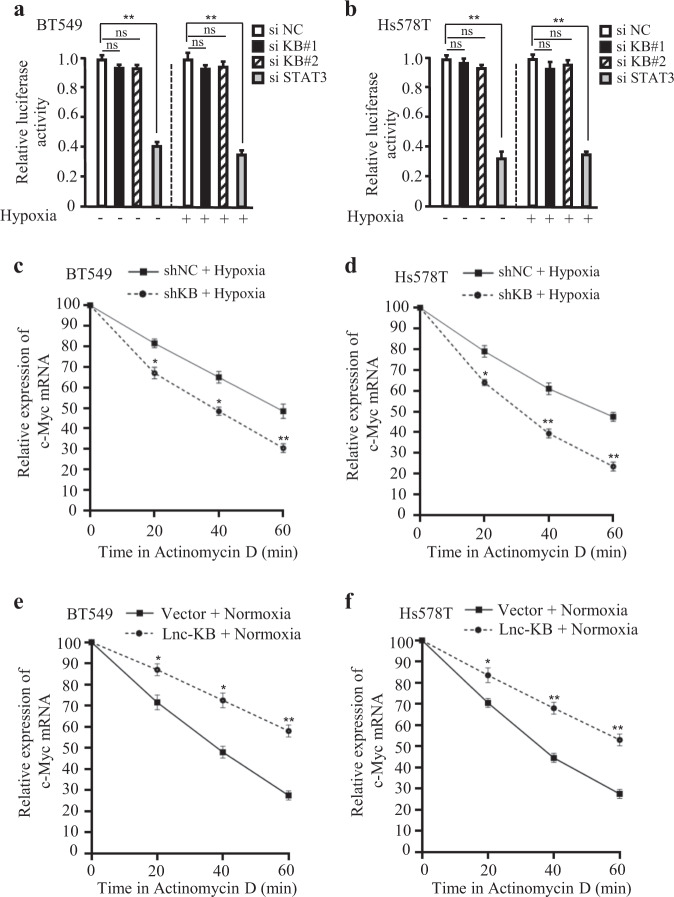
LncRNA KB-1980E6.3 increases c-Myc levels by stabilizing its mRNA. **a**, **b** Luciferase reporter assay showing the transcription activity of c-Myc in lncRNA KB-1980E6.3 knockdown and the control breast cancer cells under normoxia or hypoxia conditions. STAT3 knockdown was used as a positive control. **c**, **d** qRT-PCR was used to determine c-Myc mRNA levels in lncRNA KB-1980E6.3 knockdown and the control breast cancer cells treated with Actinomycin D for the indicated time under hypoxia conditions. **e**, **f** qRT-PCR was used to determine c-Myc mRNA levels in lncRNA KB-1980E6.3 overexpressing and the control breast cancer cells treated with Actinomycin D for the indicated time under normoxia conditions. Data are shown as the mean ± SD of three independent experiments. (ns no significance; *P < 0.05; **P < 0.01).

To understand whether lncRNA KB-1980E6.3 could impact c-Myc expression at the transcriptional level, luciferase reporter assay was conducted. The data showed that lncRNA KB-1980E6.3 knockdown had no impact on the promoter activity of c-Myc in normoxic or hypoxic breast cancer cells, however, the transcription potential of c-Myc, a known target of STAT3, was reduced in STAT3 silencing group (Fig. 5a, b), indicating that lncRNA KB-1980E6.3 may regulate c-Myc at the posttranscriptional level. We then tested c-Myc mRNA stability under treatment of Actinomycin D. Indeed, knockdown of lncRNA KB-1980E6.3 resulted in a reduced half-life of c-Myc mRNA (Fig. 5c, d), and ectopic lncRNA KB-1980E6.3 could notably increase the half-life of c-Myc mRNA (Fig. 5e, f). These data reveal that lncRNA KB-1980E6.3 closely governs c-Myc expression through stabilizing c-Myc mRNA.

### LncRNA KB-1980E6.3 increases c-Myc mRNAs stability via binding with m6A reader IGF2BP1

**Fig. 6:**
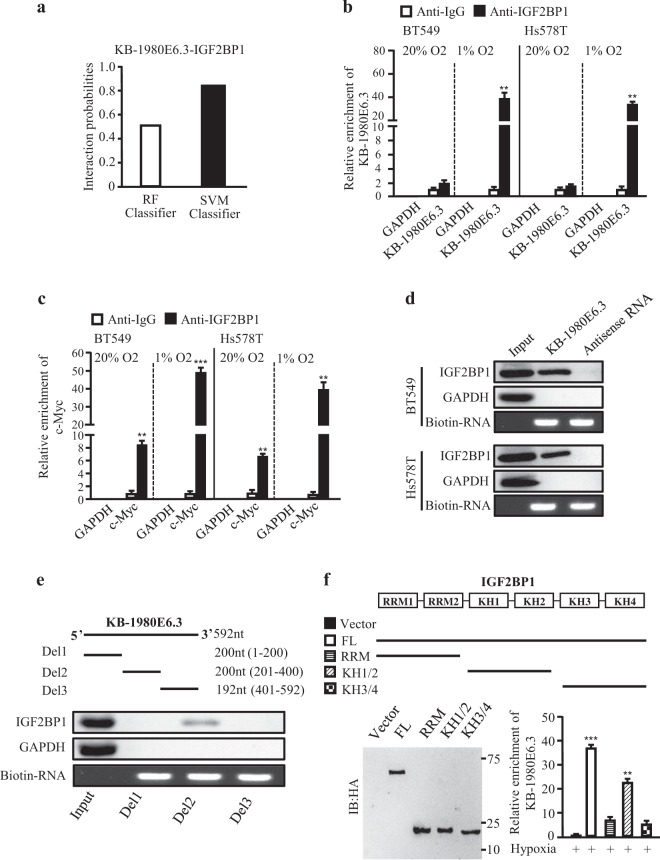
LncRNA KB-1980E6.3 increases the stability of c-Myc mRNA through binding with IGF2BP1. **a** The probability of the interaction between lncRNA KB-1980E6.3 and IGF2BP1 was predicted by RNA-Protein interaction prediction (RPISeq). **b** RIP assay was performed using cell lysates from normoxic or hypoxic breast cancer cells using anti-IGF2BP1 antibody. IgG served as a negative control. The lncRNA KB-1980E6.3 enrichment in the RIP precipitates was analyzed by qRT-PCR. GAPDH mRNA served as a negative control. **c** The enriched c-Myc in the RIP precipitates derived from normoxic and hypoxic BT549 and Hs578T cells was analyzed by qRT-PCR. GAPDH mRNA served as a negative control. **d** Lysates from hypoxic BT549 and Hs578T cells were subjected to RNA-pulldown with biotin-labeled lncRNA KB-1980E6.3 sense or antisense probe, followed by western blotting with anti-IGF2BP1 antibody. GAPDH was used as a negative control. **e** Top, schematic diagrams of lncRNA KB-1980E6.3 full-length and truncated fragments. Bottom, the interaction between lncRNA KB-1980E6.3 truncates and IGF2BP1 in hypoxic BT549 cells was examined by western blotting. **f** Top, diagrams of full-length and truncated fragments of IGF2BP1. Bottom, the immunoprecipitation efficiency of HA-tagged full-length or truncated IGF2BP1 in RIP assays (left panel). RIP-qPCR was used to identify the lncRNA KB-1980E6.3 binding domain in IGF2BP1 using full-length or truncated IGF2BP1 protein (right panel). Data are shown as mean ± SD of three independent experiments (**P < 0.01; ***P < 0.001).

Next, we asked how lncRNA KB-1980E6.3 affects c-Myc mRNAs stability. The aforementioned data showed that lncRNA KB-1980E6.3 was mainly located in the cytoplasm of hypoxic BT549 and Hs578T cells (Fig. S1e), indicating that lncRNA KB-1980E6.3 could serve as a scaffold to involve in posttranscriptional regulation of c-Myc mRNAs by directly interacting with a specific RNA-binding protein (RBP), as has been revealed for other lncRNAs [29, 30]. Thus, public bio-informatics resources, such as RNA-Protein interaction prediction (RPISeq) website, were used. After careful analysis using RPISeq, we found that IGF2BP1 was the potential RBP, binding with lncRNA KB-1980E6.3. The scores predicted by Random Forests (RF) Classifier and Support Vector Machine (SVM) Classifier were 0.5 and 0.85, respectively (Fig. 6a), suggesting a potential interaction between lncRNA KB-1980E6.3 and IGF2BP1. Through review of previous work, it was suggestive that IGF2BP1 could work as an mRNA stabilizing RBP [25, 31]. To confirm the interaction of lncRNA KB-1980E6.3 with IGF2BP1, RNA immunoprecipitation (RIP) assay was carried out using an antibody directly against IGF2BP1. A significant enrichment of lncRNA KB-1980E6.3 with IGF2BP1 was identified under hypoxic BT549 and Hs578T cells (Fig. 6b). Interestingly, c-Myc mRNA was also clearly enriched in IGF2BP1 immuno-precipitates under hypoxia conditions (Fig. 6c). We next performed RNA pull-down followed by western blotting with IGF2BP1 antibodies to further confirm the interaction between IGF2BP1 and lncRNA KB-1980E6.3. As shown in Fig. 6d, IGF2BP1 was co-precipitated with synthesized sense lncRNA KB-1980E6.3 rather than antisense lncRNA KB-1980E6.3 in hypoxic BT549 and Hs578T cells. In order to understand which fragment is necessary for the interaction between lncRNA KB-1980E6.3 and IGF2BP1, mapping assay was employed. Our data showed that the second region (201–400 nt) of lncRNA KB-1980E6.3 was required for its interaction with IGF2BP1 (Fig. 6e). IGF2BP1 is a canonical RBP, including two RNA recognition motifs (RRM) and four K homology (KH) domains, in which the KH1/2 domain is necessary for stabilization of IGF2BP-RNA complexes, and the KH3/4 domain is essential for its binding function with target RNA [32]. To investigate which domain of IGF2BP1 might play a key role in interacting with lncRNA KB-1980E6.3, RIP assays using antibodies against HA-tagged full-length or truncated IGF2BP1 (RRM, KH domains) were carried out. The results showed that the KH1/2 domain of IGF2BP1 was required for association with lncRNA KB-1980E6.3 in hypoxic BT549 cells (Fig. 6f).

Previous studies have shown a control of IGF2BP1 on c-Myc mRNA stability under normoxia [33]. To further validate the result in breast cancer cells, the IGF2BP1 was knocked down or overexpressed in BT549 and Hs578T cells. The results showed that knockdown of IGF2BP1 led to reduced c-Myc mRNA stability, and ectopic IGF2BP1 could further increase c-Myc mRNA stability in normoxic breast cancer cells (Fig. S7a–d). To further confirm the stability of c-Myc mRNA is associated with lncRNA KB-1980E6.3 mediated IGF2BP1 recruitment under hypoxia conditions, we then performed a rescue experiment in lncRNA KB-1980E6.3 knockdown tumor cells and found that the decreased mRNA stabilities, mRNA and protein of c-Myc caused by lncRNA KB-1980E6.3 knockdown were partially restored by ectopic IGF2BP1 expression in hypoxic BT549 and Hs578T cells (Fig. S7e–h). This suggested that lncRNA KB-1980E6.3 could recruit IGF2BP1 to retain c-Myc mRNA stability in hypoxic breast cancer cells. In addition, endogenous IGF2BP1 levels remained the same in lncRNA KB-1980E6.3 knockdown breast cancer cells under nomorxia and hypoxia (Fig. S7i, j). Thus, a low ratio of lncRNA KB-1980E6.3/IGF2BP1 was found under normoxia, and hypoxia-induced high level of lncRNA KB-1980E6.3 resulted in a high lncRNA KB-1980E6.3/IGF2BP1 ratio under hypoxia, which was reduced in accompany with lncRNA KB-1980E6.3 knockdown in hypoxic breast cancer cells (Fig. S7k). These data indicate that IGF2BP1 is not the target of lncRNA KB-1980E6.3, and c-Myc mRNA stability is dependent on lncRNA KB-1980E6.3 mediated recruitment of IGF2BP1 rather than hypoxia-regulated IGF2BP1 expression.

**Fig. 7:**
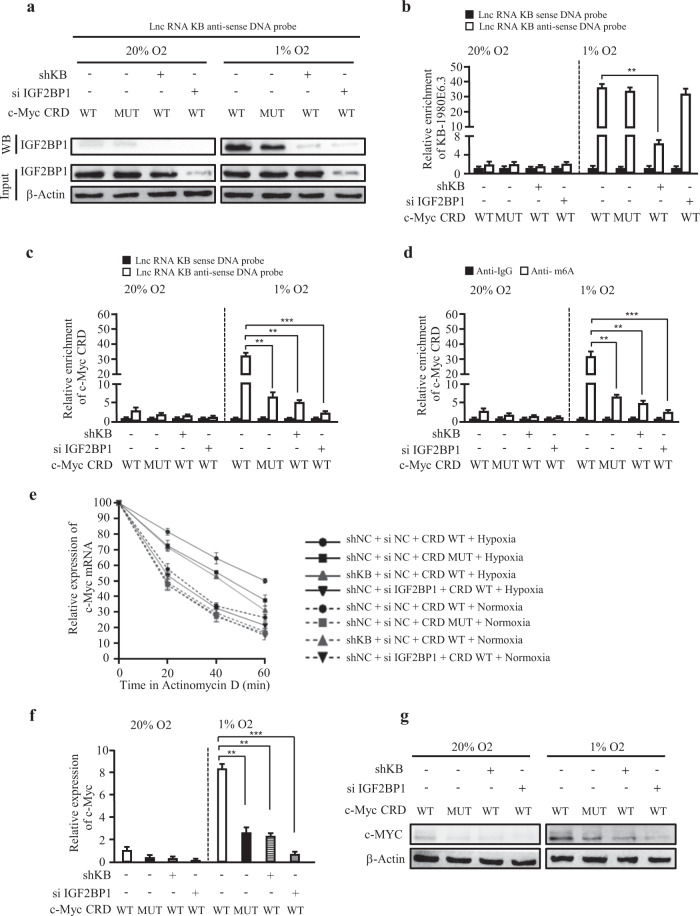
LncRNA KB-1980E6.3 increases the stability of c-Myc mRNA through recruitment of IGF2BP1 to bind with m6A-modified c-Myc CRD mRNA. **a**, **b** The plasmid of c-Myc wild type CRD (WT) or mutated CRD (MUT) was co-transfected with or without siIGF2BP1 into endogenous c-Myc knockdown BT549 cells or c-Myc and lncRNA KB-1980E6.3 double knockdown BT549 cells, respectively. The indicated BT549 lysates were incubated with either sense or antisense biotin-labeled probe against lncRNA KB-1980E6.3 for the RNA pull-down assay. Western blotting was used to detect the IGF2BP1 protein (**a**), and qRT-PCR was used to detect lncRNA KB-1980E6.3 (**b**) in the pull-down precipitates. **c** qRT-PCR was used to determine the enrichment of c-Myc CRD mRNA by lncRNA KB-1980E6.3-recruited IGF2BP1 in the pull-down precipitates. **d** Gene-specific m6A qPCR assays were performed in cellular lysates from the indicated engineered cells described in (**a**) to detect the m6A-modified c-Myc CRD mRNA levels. **e** The indicated engineered BT549 cells described in (**a**) were treated with Actinomycin D for the indicated times, and c-Myc mRNA levels were measured by qRT-PCR. **f**, **g** c-Myc mRNA and protein levels were examined in the engineered cells described in (**a**) by qRT-PCR and western blotting. Data are shown as mean ± SD of three independent experiments (**P < 0.01; ***P < 0.001).

Finally, we asked why the interaction of IGF2BP1 with lncRNA KB-1980E6.3 is essential for c-Myc mRNA stability. It was reported that m6A methylation impacted mRNA stability and that IGF2BP1 could serve as m6A-reader in recognition of mRNA m6A methylation [25]. Studies have shown that there is a ~250 nucleotide *cis*-acting element, named coding region instability determinant (CRD), in the 3′-terminus of c-Myc mRNA coding region that is vital for IGF2BP1 binding; and high m6A modifications in the CRD region are helpful to c-Myc stability [25, 33]. The c-Myc CRD has six m6A consensus sequences (–GGACT–, –GAACA–, –AAACA–, –AAACT–, –GAACA–, –GAACT–; WT). We mutated those m6A sites to obtain a c-Myc CRD mutant construct (–GGTCT–, –GATCA–, –AATCA–, –AATCT–, –GATCA–, –GATCT–; mutant, MUT), then the c-Myc CRD WT or c-Myc CRD MUT plasmid was co-transfected with or without siIGF2BP1 into endogenous c-Myc knockdown BT549 cells or endogenous c-Myc and lncRNA KB-1980E6.3, doubly silencing BT549 cells, respectively. Then using a biotin-labeled antisense DNA probe specifically against lncRNA KB-1980E6.3 in pull-down assay, we found that IGF2BP1 and lncRNA KB-1980E6.3 were co-enriched in the pull-down precipitates of hypoxic BT549 cells confirmed by western blotting and qRT-PCR; the loss of IGF2BP1 or lncRNA KB-1980E6.3 notably decreased IGF2BP1 and lncRNA KB-1980E6.3 levels in the pull-down precipitates (Fig. 7a, b), suggesting IGF2BP1 could be recruited by lncRNA KB-1980E6.3. To understand whether lncRNA KB-1980E6.3-mediated recruitment of IGF2BP1 could facilitate recognition between IGF2BP1 and c-Myc CRD mRNA under hypoxia, we detected the enrichment of c-Myc CRD mRNA in the pull-down precipitates. Indeed, the recruited IGF2BP1 by lncRNA KB-1980E6.3 could bind significantly more with c-Myc CRD WT than c-Myc CRD mutant under hypoxia (Fig. 7c), indicating a lncRNA KB-1980E6.3-mediated recognition between IGF2BP1 and c-Myc CRD mRNA in hypoxic BT549 cells. Knockdown of lncRNA KB-1980E6.3 or IGF2BP1 markedly reduced the recognition between IGF2BP1 and c-Myc CRD mRNA in hypoxic BT549 cells (Fig. 7c). In addition, using gene-specific m6A qPCR, we detected more m6A-modified c-Myc CRD mRNA in the precipitates derived from hypoxic BT549 under lncRNA KB-1980E6.3/IGF2BP1 complex binding with WT c-Myc CRD; the loss of lncRNA KB-1980E6.3 or IGF2BP1, or transfection of c-Myc CRD mutant, significantly decreased m6A-modified c-Myc CRD mRNA in hypoxic BT549 cells (Fig. 7d).

We next detected whether lncRNA KB-1980E6.3/IGF2BP1-mediated m6A methylation in c-Myc CRD could play a key role in regulating c-Myc mRNA stability. As shown in Fig. 7e, significantly stable c-Myc mRNA was detected in hypoxic BT549 cells with ectopic WT c-Myc CRD rather than mutant c-Myc CRD; the loss of lncRNA KB-1980E6.3 or IGF2BP1 dramatically decreased the stability of c-Myc mRNA in hypoxic tumor cells. In contrast, there was weak c-Myc stability in normoxic BT549 cells. Correspondingly, high levels of c-Myc mRNA and protein were detected in BT549 cells with ectopic WT c-Myc CRD in compared with those of any other groups under hypoxia conditions (Fig. 7f, g). These data demonstrate that lncRNA KB-1980E6.3 increases the binding of IGF2BP1 with m6A-modified c-Myc CRD mRNA and leads to the stability of c-Myc mRNA in hypoxic breast cancers.

### LncRNA KB-1980E6.3-mediated c-Myc stability is essential for BCSCs stemness and tumorigenesis in vivo

**Fig. 8:**
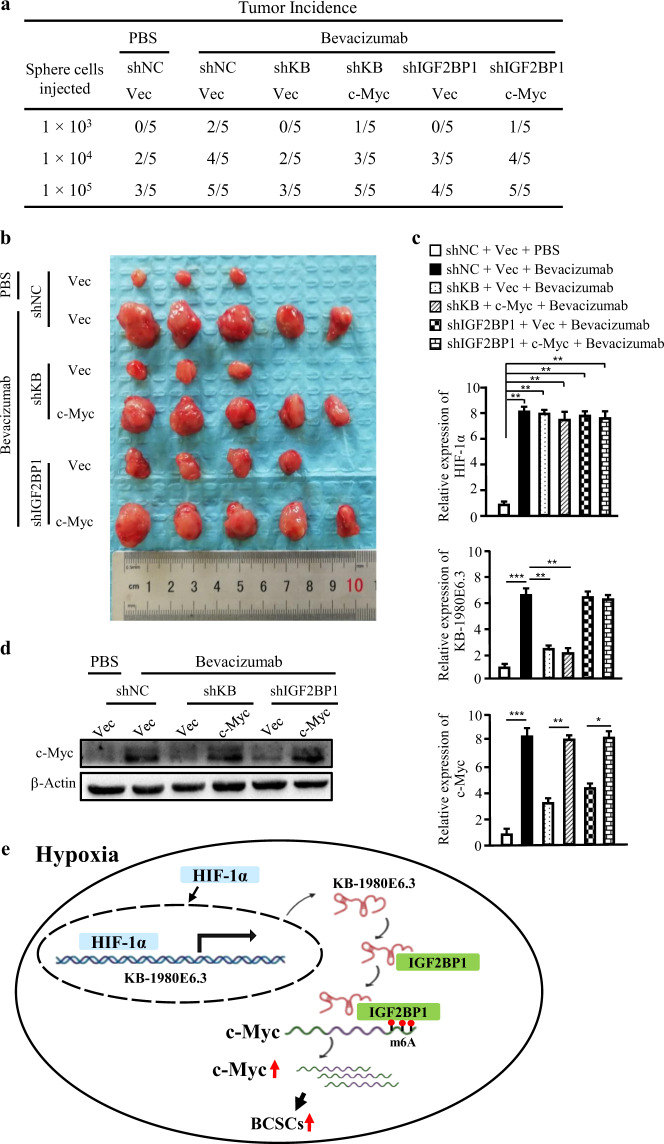
LncRNA KB-1980E6.3-mediated c-Myc stability is essential for tumorigenesis of BCSCs in vivo. The indicated sphere cells (1 × 10^5^, 1 × 10^4^ and 1 × 10^3^) derived from engineered Hs578T including Hs578T/shKB/vector, Hs578T/shKB/c-Myc, Hs578T/shIGF2BP1/vector, Hs578T/shIGF2BP1/c-Myc, and Hs578T/shNC/vector control cells were injected into nude mice, and mice were administrated with bevacizumab at 10 mg/kg or vehicle (PBS). **a** Tumor incidence in each treatment group. **b** Representative images of tumors in nude mice injected with 1 × 10^5^ sphere cells derived from engineering breast cancer cells described above. **c** Expression levels of HIF-1α, lncRNA KB-1980E6.3, and c-Myc in mouse xenograft tumors were detected by qRT-PCR. **d** c-Myc proteins in mouse xenograft tumor tissues were detected by western blotting. **e** Schematic diagram showing that HIF-1α induces lncRNA KB-1980E6.3 to recruit IGF2BP1 to form lncRNA KB-1980E6.3/IGF2BP1 complex, which in turn recognizes m6A-modified c-Myc CRD mRNA and enhances c-Myc mRNA stability and eventually maintain BCSCs stemness and tumor progression under a hypoxic tumor microenvironment. Data are shown as mean ± SD of three independent experiments (*P < 0.05; **P < 0.01; ***P < 0.001).

To understand whether hypoxic lncRNA KB-1980E6.3-mediated c-Myc stabilit**y** is pivotal in maintaining BCSCs stemness, we investigated the CSC stemness of hypoxic BT549 and Hs578T under interfering lncRNA KB-1980E6.3/IGF2BP1/c-Myc complex. Indeed, decreased spheroid formation capacity and cell colony formation under lncRNA KB-1980E6.3 or IGF2BP1 depletion were observed and could be partially rescued by ectopic c-Myc expression in hypoxic BT549 and Hs578T cells (Fig. S8a–e). Next, we explored whether lncRNA KB-1980E6.3/IGF2BP1/c-Myc complex could contribute to BCSCs-derived tumor initiation and tumor growth in vivo. Three doses (1 × 10^5^, 1 × 10^4^, and 1 × 10^3^) of spheres derived from the engineered Hs578T and 1 × 10^5^ of spheres derived from the engineered BT549, including shKB/vector, shKB/c-Myc, shIGF2BP1/vector, shIGF2BP1/c-Myc, and shNC/vector control cells, were subcutaneously inoculated into 4- to 6-week-old female nude mice (*n* = 5 per group), respectively. Mice were then treated with either bevacizumab to form a hypoxic tumor microenvironment or PBS to form a non-hypoxic condition. Bevacizumab-induced hypoxic microenvironment in mouse tumor increased tumorigenesis and tumor growth compared with PBS administrated xenografts (Figs. 8a, b, and S9a, b). Knockdown of lncRNA KB-1980E6.3 or IGF2BP1 in Hs578T CSCs significantly decreased tumor initiation and tumor growth in comparison with their control groups; however, ectopic c-Myc could effectively rescue tumor initiation and tumor growth caused by lncRNA KB-1980E6.3 silencing or IGF2BP1 knockdown in bevacizumab-administrated mice (Figs. 8a, b, and S9a, b). Correspondingly, high levels of HIF-1α, lncRNA KB-1980E6.3, and c-Myc were efficiently induced in bevacizumab-administrated tumors compared with control tumors (Fig. 8c, d). Silence of lncRNA KB-1980E6.3 or IGF2BP1 led to reduction of c-Myc (Figs. 8c, d, and S9c) and CD44 biomarker of BCSCs in hypoxic tumors (Fig. S9c), which were consistent with reduced tumorigenesis incidence (Fig. 8a, b). This conclusion was further supported by using the indicated engineered BT549 CSCs (Fig. S10). Taken together, these data indicate that hypoxia-induced lncRNA KB-1980E6.3/IGF2BP1/c-Myc axis is essential for the maintenance of BCSCs, thus leading to tumor initiation and tumor growth in vivo (Fig. 8e).

## Discussion

Hypoxic microenvironments are characteristic of rapidly growing tumors, which profoundly impact tumor progression across an array of cancer types. The cellular response to hypoxia insult is mainly governed by HIF. HIF can stabilize and activate the transcription of specific target genes by binding to HRE which contains the core sequence 5-(A/G) CGTG-3 under hypoxia conditions. In addition to being a protein-coding gene, a fair percentage of lncRNAs can also be regulated in response to hypoxia. Indeed, the network of HRLs and their downstream targets have demonstrated to offer an exquisite coordination under hypoxia conditions. Accordingly, those HRLs, functioning as oncogenes or anti-oncogenes, have been reported to play central roles in tumorigenesis, metastasis, and the prognosis of various solid cancers. For example, lincRNAp21, an HRL, is essential for hypoxia-enhanced glycolysis [34]. CF129, another HRL, is closely related to multiple clinicopathologic characteristics of pancreatic cancer patients [35]. However, the biological roles of HRLs in breast cancer have yet to be understood mechanistically.

In the current study, we identified lncRNA KB-1980E6.3 as a new player for breast cancer cells’ response to hypoxia. Notably, lncRNA KB-1980E6.3 is upregulated in breast cancer cells, and breast cancer patients with high level of lncRNA KB-1980E6.3 demonstrated an adverse prognosis. Similar with most HIF-dependent HRLs, HIF-1α can directly bind with the HRE in lncRNA KB-1980E6.3 promoter to regulate its transcription activity under hypoxia. Moreover, lncRNA KB-1980E6.3 plays an essential role for BCSCs stemness by enhancing c-Myc mRNA stability via interaction with IGF2BP1, which may provide breast cancer cells with more flexibility to adapt to hypoxic environmental conditions.

Of note, lncRNA KB-1980E6.3 participates in regulating posttranscriptional events. In fact, HRLs can utilize multiple mechanisms to control the fine-tuning of hypoxia-induced processes. Some HRLs modulate hypoxic gene expression by epigenetic regulatory mechanism. For example, lncRNA-AK058003, could be robustly induced by hypoxia, facilitates gastric cancer cell migration and invasion through DNA demethylation of SNCG gene [36]. In addition, some HRLs can fine-tune hypoxia networks in a transcription regulatory way. As reported, hypoxia-induced lncRNA-BX111 promoted metastasis and progression of pancreatic cancer through regulating ZEB1 transcription [37]. In particular, some HRLs are subjected to a series of posttranscriptional modifications to control gene expression, such as lncRNA UCA1, which can act as a competitive endogenous RNA (ceRNAs) and is involved in miRNA-mediated mRNA destabilization as an endogenous miRNA ‘sponge’ or ‘decoy’ in hypoxia-resistant gastric cancer cells [38]. In addition, hypoxia-induced lncRNA DARS-AS1 exerts its function by binding RNA-binding motif protein 39 (RBM39), which impedes the interaction between RBM39 and its E3 ubiquitin ligase RNF147, to prevent RBM39 degradation [39]. Notably, our current work reveals that lncRNA KB-1980E6.3 is involved in posttranscriptional modulation of c-Myc gene by stabilizing c-Myc mRNA, which provides a novel orchestrated regulatory network of HRLs in the control of cancer-related events under hypoxia.

LncRNA KB-1980E6.3 could recruit IGF2BP1 to regulate c-Myc mRNA stability at the posttranscriptional level. Emerging evidence indicates that lncRNAs can regulate mRNA stability through various mechanisms. First, lncRNAs could enhance mRNA stability by directly binding to the 3′-untranslated regions (UTR) of target genes. For example, lncRNA THOR can directly bind to the 3′UTR of SOX9, thereby enhancing SOX9 mRNA stability, and promote the stemness of gastric cancer cells and osteosarcoma cells [40, 41]. Second, lncRNAs could regulate mRNA stability via indirectly interacting with RBPs. For example, lncRNA RMST can increase DNMT3B stability by enhancing the interaction between DNMT3B and HuR [42]. Significantly, lncRNAs could competitively combine with RBPs that have a stabilizational or destabilizational effect on target genes. For example, linc-RoR could interact with heterogeneous nuclear ribonucleoprotein (hnRNP) I (a stabilizing factor) and enhance c-Myc mRNA stability, which is mostly due to its competition with AUF1 (a destabilizing factor) for c-Myc mRNA [43]. Our work unveils that hypoxia-stimulated lncRNA KB-1980E6.3 recruits IGF2BP1 to regulate c-Myc mRNA stability via binding with m6A-modified c-Myc CRD mRNA.

Increasing evidence suggest that lncRNAs share microRNA recognition elements (MREs) with specific mRNAs, in which lncRNAs act as ceRNA by functioning as decoys of microRNA, thus regulating the expression of target mRNAs. Those ceRNA mechanisms play a central role in promoting the initiation and progression of various cancers [44–46]. As lncRNA KB-1980E6.3 is highly expressed in breast cancer and mainly locates in the cytoplasm of hypoxic BT549 and Hs578T cells, we wonder whether lncRNA KB-1980E6.3 could regulate the expression of some oncogenes via acting as a ceRNA under hypoxia conditions. This speculation and the detailed mechanism should be explored in future work.

LncRNA KB-1980E6.3 increases c-Myc mRNAs stability via binding with m6A reader IGF2BP1. IGF2BP1 is composed of six canonical RNA-binding domains, including two RRM domains and four KH domains. It has been reported that IGF2BP1 has a potent binding ability to some well-known mRNA targets, including IGF2, c-Myc, ACTIN, PTEN, and CTNNB1, to regulate their mRNA stability [33, 47, 48]. Emerging evidence have highlighted that a number of lncRNAs play a fine-tuning role in modulating the interactions between IGF2BP1 and their target genes [49–51]. Consistent with these findings, our data showed that hypoxia-induced lncRNA KB-1980E6.3 primarily bound to the KH1/2 domain of IGF2BP1, leading to an increase in the binding ability of IGF2BP1 to m6A-modified c-Myc CRD mRNA and maintenance of c-Myc mRNA stability in breast cancer cells.

LncRNA KB-1980E6.3 is essential for the enhanced stemness of breast cancer cells in a hypoxic microenvironment. Hypoxia seems to promote a specific environment that makes it easier for the most robust clone to evolve hierarchically and grow rapidly. Remarkably, hypoxia or hypoxia-sensing pathways play a significant role in the maintenance of BCSCs phenotypes through activation of target pluripotency factors by HIF. For example, HIF-dependent ALKBH5 expression mediates enrichment of BCSCs in the hypoxic tumor microenvironment [52]. HIF-1α-dependent expression of adenosine receptor 2B promotes BCSCs enrichment [53]. Our data further underscore the important role of the hypoxic niche on BCSCs properties.

We confirm a pivotal role of lncRNA KB-1980E6.3 in BCSCs stemness maintenance by regulating c-Myc. c-Myc, as a master regulator, is well known for its function in maintaining self-renewal of CSCs in several malignancies. In recent years, it has become clear that multilayered microRNAs and lncRNAs can influence the expression level of c-Myc by regulating its transcription, translation, and activity. Some microRNAs can regulate c-Myc by binding to its 3′UTR, 5′UTR, and protein-coding sequence [54–56]. In addition, other microRNAs can influence the protein level of c-Myc by interacting with different target genes [57, 58]. And, some microRNAs can directly regulate c-Myc by binding to c-Myc mRNA [59]. Notably, numerous lncRNAs modulate the expression level of c-Myc through various mechanism. They can regulate the transcription of c-Myc in *cis*, control mRNA stability and translation of c-Myc, or affect protein stability and activity of c-Myc [60–64]. The current work expands our understanding on those special HRLs, which play a powerful role in controlling CSCs characteristics by regulating stemness related transcription factors in a particular hypoxic environment.

In conclusion, hypoxia-induced lncRNA KB-1980E6.3 is involved in the self-renewal and stemness maintenance of BCSCs by recruiting IGF2BP1 to regulate c-Myc mRNA stability. The newly identified lncRNA KB-1980E6.3/IGF2BP1/c-Myc axis may potentially be a therapeutic target for breast cancer.

## Materials and methods

### Cell culture, RNA interference and plasmids

Human breast cancer cells (BT549, MDA-MB-231, MCF-7, Hs578T, T47D, MDA-MB-453, BT474, and MDA-MB-468), and HEK293T embryonic kidney cells were acquired from the American Type Culture Collection (ATCC). These cells were cultured in RPMI 1640 or DMEM medium (Gibco-BRL, Australia) containing 10% fetal bovine serum (Gibco-BRL, Australia) at 37 °C in humidified atmosphere containing 5% CO_2_ with 1% O_2_ (Hypoxia condition) or 20% O_2_ (Normoxia condition).

The small interfering RNA (siRNAs) specifically against HIF-1α, HIF-2α, IGF2BP1, c-Myc, lncRNA KB-1980E6.3 (GenePharama, Shanghai, China) were used to transiently knockdown target genes in breast cancer cells with Lipofectamine 3000 (Invitrogen, USA) following the manufacturer’s instructions. To establish the stably interfered or target gene expressed cells, lentivirus-mediated shRNA specifically against HIF-1α (shHIF-1α), HIF-2α (shHIF-2α), lncRNA KB-1980E6.3 (shKB), or IGF2BP1 (shIGF2BP1), and lentiviral of lncRNA KB-1980E6.3 overexpressing construct (GenePharama, Shanghai, China) were respectively transfected into breast cancer cells according to the manufacturer’s protocols. Positively infected cells were selected with puromycin treatment and the selected cell pool was used in experiments.

The lncRNA KB-1980E6.3 promoter containing HIF-binding sites (WT: –GCGTG–) and its mutant sites (MUT: –GGCAG–) were cloned into a pGL3 luciferase reporter vector to obtain the pGL3-lncRNA KB-1980E6.3 WT reporter or pGL3-lncRNA KB-1980E6.3 MUT reporter. The promoter of c-Myc was cloned into pGL3 luciferase reporter vector to construct the pGL3-c-Myc reporter. The pcDNA3.3/IGF2BP1 (WT), pcDNA3.3/IGF2BP1-RRM domains, pcDNA3.3/IGF2BP1-KH domains, pcDNA3.3/c-Myc, pcDNA3.3/HIF-1α, and pcDNA3.3/HIF-2α plasmids were created by PCR and inserted into a pcDNA3.3 vector. The sequences of siRNAs and shRNA used in this study have been listed in Supplementary Table 2 and Supplementary Table 3.

### RNA preparation and quantitative real-time PCR (qRT-PCR)

TRIZOL reagent (Invitrogen) was used to extract total RNA in both tissues and cells according to the manufacturer’s instruction. The cDNA was obtained from the purified RNA using a PrimeScript RT Reagent Kit (Takara). SYBR Premix Ex Taq II Kit (Takara) was used for qRT-PCR assays. Results were normalized to β-actin expression. All experiments were performed at least three times. All specific primers used in this study have been listed in Supplementary Table 4.

### Tissue samples

Human breast tumor tissues and their corresponding normal breast tissues were obtained from the First Affiliated Hospital of Chongqing Medical University, and the experiments were approved by the Research Ethics Committee of Chongqing Medical University. All patients did not receive any radiotherapy or chemotherapy previously. All patients have been informed and consented involving this study.

### Mammosphere formation assay

BCSCs were cultured as described previously [65]. Briefly, breast cancer cells were dissociated into single cells by 0.05% trypsin-EDTA solution and plated into six-well plates coated with 2% poly-HEMA (Sigma) at a density of 1 × 10^4^ cells/ml in primary culture and 5 × 10^3^ cells/ml in following passages in normoxic or hypoxic conditions. Mammosphere cells were enriched using serum-free medium, which was composed of DMEM-F12 medium with epidermal growth factor (EGF, 20 ng/ml), Basic fibroblast growth factor (b-FGF, 20 ng/mL), insulin (5 μg/ml), and B27 (Invitrogen). The numbers of secondary generation spheres were counted using an OLYMPUS IX70 microscope (Tokyo, Japan). The percentage of mammosphere forming efficiency (MFE) was calculated as previously described. The average size of the randomly selected mammospheres (*N* = 30) was calculated.

### TCGA database analysis

TCGA database is a large cancer database that integrates the global gene expression profiles between multiple tumors and their corresponding non-tumor tissues, containing genomic sequencing data of more than 20 kinds of human tumors [66]. In our analysis, an RNA-seq data with clinical information of 1100 breast cancer specimens and 113 normal specimens were scanned and extracted from the cohort: GDC TCGA Breast Cancer (https://portal.gdc.cancer.gov/). The expression trend of lncRNA KB-1980E6.3 was determined by RNA sequencing as Fragments per kilobase of exon model per million mapped reads (FPKM).

### In vivo Xenograft experiments

Animal experiments were performed in accordance with guidelines on animal care approved by the Chongqing Medical University Experimental Animal Management Committee. The enriched mammosphere cells derived from engineered BT549 and Hs578T with silenced lncRNA KB-1980E6.3 (shKB/vector), BT549, and Hs578T with lncRNA KB-1980E6.3 knockdown combined with ectopic c-Myc (shKB/c-Myc), BT549, and Hs578T with silenced IGF2BP1 (shIGF2BP1/vector), BT549, and Hs578T with knocked down IGF2BP1 combined with ectopic c-Myc (shIGF2BP1/c-Myc), and BT549, and Hs578T/shNC/vector control cells were used in Xenograft experiments. Three doses (1 × 10^5^, 1 × 10^4^ and 1 × 10^3^) of spheres derived from the engineered Hs578T and 1 × 10^5^ of spheres derived from the engineered BT549 were subcutaneously inoculated into 4- to 6-week-old female nude mice (*n* = 5 per group). Mice were then treated with either bevacizumab (10 mg/kg every 3 days) to form a hypoxic tumor microenvironment or vehicle PBS to form a non-hypoxic condition [67, 68]. The tumor-initiation frequency was calculated. The longest diameter and its widest vertical width of tumor were measured every 3 days with a dialcaliper, and tumor volume was calculated by the equation (*V* = length × width^2^ × 0.5). At the end of animal experiments, xenografted mice were sacrificed and the tumor tissues were surgically removed, measured, and weighed. The acquired tumor tissues were subjected to qRT-PCR, western blotting or immunohistochemistry staining to detect HIF-1α, lncRNA KB-1980E6.3, c-Myc, and CD44 expressions.

### Statistical analysis

Statistical analyses were conducted with the employment of SPSS 20.0 statistical software and GraphPad Prism 7.0 (GraphPad Software, Inc., La Jolla, CA, USA). To assess comparisons between multiple groups, ANOVA followed by the Student–Newman–Keuls multiple comparisons test was performed. To assess comparisons between two groups, the Student’s *t* test was used. The correlation between groups was analyzed by Pearson correlation. Each experiment was performed thrice and data were shown as mean ± SD. Any value of *P* < 0.05 was considered to be statistically significant.

### Additional materials and method

The details of other materials and methods can been saw at the Supplementary material.

## Supplementary information

Supplementary materials

Suppl Figures

Supplementary Figure legends

Supplementary Table 1

Supplementary Table 2

Supplementary Table 3

Supplementary Table 4
