# Supplementary material for: A novel hypoxic long noncoding RNA KB-1980E6.3 maintains breast cancer stem cell stemness via interacting with IGF2BP1 to facilitate c-Myc mRNA stability
Source: Oncogene. 2021 Jan 19;40(9):1609–27. doi: 10.1038/s41388-020-01638-9 (PMC7932928; doi:10.1038/s41388-020-01638-9)
Supplement: Supplementary file 1 — Supplementary materials [file 41388_2020_1638_MOESM1_ESM.docx]

Supplementary materials

Luciferase assay

HEK293T cells were transfected with pGL3-lncRNA KB-1980E6.3 WT reporter, pGL3-lncRNA KB-1980E6.3 MUT reporter or control pGL3 reporter and pRL-TK constructor using Lipofectamine 3000, and then co-transfected with (0, 25, 50 and 100 ng) HIF-1α or HIF-2α plasmid. The corresponding wells were then cultured under normoxic conditions for 24 hours. Breast cancer cells were transfected with pGL3-lncRNA KB-1980E6.3 WT reporter or pGL3-KB-1980E6.3 MUT reporter combined with pRL-TK constructor using Lipofectamine 3000 and then transfected with siHIF-1α or siHIF-2α. The corresponding wells were cultured under hypoxic conditions. Similarly, breast cancer cells were transfected with pGL3-c-Myc reporter and pRL-TK constructor using Lipofectamine 3000 and then transfected with siKB or siSTAT3. The corresponding wells were cultured under normoxia or hypoxia. Luciferase activities were detected with a Dual-Luciferase Reporter System (Promega, USA), and normalized by using pRL-TK reporters as an internal control. The experiment was performed in triplicate.

Chromatin immunoprecipitation (ChIP)

ChIP assay was conducted using the EZ-ChIP^TM^ Chromatin Immunoprecipitation Kit (Millipore, Billerica, MA, USA) according to the manufacturer's instructions. The anti-HIF-1α or HIF-2α antibody and corresponding IgG were used to pull down the target protein. The eluted DNA was amplified by PCR, and then analyzed via 2% agarose gel electrophoresis. Primer sequences are given in Supplementary Table 4. All ChIP assays were repeated independently three times.

Colony formation assay

For colony formation assay, breast cancer cells (1×10^3^ cells/well) were seeded into 6‐well plates and cultured in RPMI 1640 medium in normoxic or hypoxic conditions. After 14 days, cells were washed with PBS and fixed with methanol for 15 min and stained with 0.5% crystal violet for 15 min. Colonies were counted under a microscope.

Western blot assay

Cell lysates were prepared using RIPA buffer (Beyotime Biotechnology, China) containing a protease inhibitor cocktail. After measuring protein concentration using BCA assay kit (Beyotime, Jiangsu, China), equal amounts of protein were separated by 8-10% sodium dodecyl sulfate polyacrylamide gel electrophoresis (SDS-PAGE) and transferred onto a polyvinylidence difluoride (PVDF) membrane. Proteins were incubated with specific primary antibodies as follows: anti-HIF-1α (1:1000, Abcam), anti-HIF-2α (1:1000, Abcam), anti-Nanog (1:1000, Abcam), anti-SOX2 (1:500, Abcam), anti-OCT4 (1:1000, Abcam), anti- KLF4 (1:500, Proteintech), anti- c-Myc (1:1000, Proteintech), anti-IGF2BP1 (1:1000, Proteintech), anti-β-actin (1:1000, Abcam). After washing for three times, the membrane was incubated with the species-matched secondary antibodies at room temperature for 2h. Finally, the antigen–antibody reaction was visualized using the enhanced chemiluminescence system (Amersham Pharmacia Biotech) and normalized to β-actin. All experiments were performed at least three times.

Immunohistochemistry (IHC) analysis

The paraffin-embedded tumor and normal tissues were sectioned at 4 μm thickness and heated for antigen retrieval at 95 °C in citric acid buffer (pH 6.0). Treated with 3% H_2_O_2_, and blocked with 5% goat normal serum, the slices were incubated with primary antibody against c-Myc (1:100, Proteintech), CD44 (1:200, Proteintech), and the corresponding secondary antibody. c-Myc and CD44 expression levels were assessed by the scores of positively labeled cells containing brown particles, which were recorded as: 0 (positive rate < 25%), 1 (positive rate: 25–50%), 2 (positive rate: 51–75%), and 3 (positive rate > 75%). IHC staining intensities (I) were scored as: 0 (negative), 1+ (weak), 2+ (moderate), 3+ (strong). Results were evaluated by two pathologists who were blinded to the experiment separately.

RNA sequence and bioinformatics analysis

The sequencing data of lncRNA KB-1980E6.3 was analyzed by the University of California Santa Cruz (UCSC) genome browser database (http://genome.ucsc.edu/). Noncoding prediction of lncRNA KB-1980E6.3 was performed by the online prediction software Coding Potential Calculator (CPC) (<http://cpc.cbi.pku.edu.cn/>) and RNA coding potential assessment tool ([CPAT](https://sourceforge.net/projects/rna-cpat/)) (http://lilab.research.bcm.edu/cpat/index.php). The interaction between lncRNA KB-1980E6.3 and IGF2BP1 was predicated by RNA-Protein interaction prediction (RPISeq) website (http://pridb.gdcb.iastate.edu/RPISeq/).

Subcellular fractionation assay of RNA

The separation of nuclear and cytosolic RNA fractions was performed using the PARIS^TM^ Kit (Invitrogen, USA) according to the manufacturer’s instructions. RNA levels of lncRNA KB-1980E6.3, U6 (nuclear control transcript), and GAPDH (cytoplasmic control transcript) were analyzed by qRT-PCR.

RNA immunoprecipitation (RIP)

RIP experiments were employed using the Magna RIP™ RNA-Binding Protein Immunoprecipitation Kit (Millipore, USA) based on the manufacturer’s instructions. Cells at 80-90% confluency were scraped off and lysed in complete RIP lysis buffer. One hundred microliters of whole cell extract were incubated with suspended beads conjugated with IGF2BP1 antibody or control IgG at 4°C overnight. After washing the beads, the bead-bound immunoprecipitate was digested with proteinase K. RNAs were purified by phenol/chloroform/isoamyl alcohol, and then subjected to qRT-PCR. The RNA levels were normalized to the input control.

Gene-specific m6A qPCR

Gene-specific m6A qPCR were employed using the Magna MeRIP m6A Kit (Millipore, Billerica, MA) based on the manufacturer’s instructions. 100 µg of total RNA was sheared to approximately 100 nt in length by metal-ion induced fragmentation and purified, then incubated with m6A antibody or control IgG-conjugated beads in 500 µL 1× IP buffer containing RNase inhibitors at 4°C overnight. Methylated RNA was immunoprecipitated with beads, eluted by competition with free m6A and recovered with the RNeasy kit (Qiagen). One tenth of fragmented RNA was saved as input control, and further analyzed by qPCR along with the MeRIPed RNAs. The related enrichment of m6A in each group were normalized to the ten-fold input.

RNA pull-down assay

The full-length sense and antisense lncRNA KB-1980E6.3 were prepared by in vitro transcription with biotin RNA labeling mix and T7 RNA polymerase (Roche, USA) based on the manufacturer’s instructions. Biotin-labeled lncRNA KB-1980E6.3 was mixed with cellular extracts of hypoxic BT549 and Hs578T cells, and afterwards, streptavidin beads were introduced. Then, the mixture was washed and eluted. The eluted solutions were analyzed by SDS-PAGE, followed by western blotting.

For antisense oligomer affinity pull-down assay, sense or antisense biotin-labeled DNA oligomers against lncRNA KB-1980E6.3 were incubated with the indicated BT549 cell lysates for two hours. Streptavidin beads were added to isolate the RNA-RNA or RNA-protein complex and then detected by qRT-PCR and western blotting.

**RNA stability assays**

Breast cancer cells were treated with Actinomycin D (2.5 µg/ml) for blocking mRNA transcription. Then cells were reaped at the indicated times points and total RNA was extracted using TRIZOL reagent (Invitrogen). c-Myc levels were measured by qRT-PCR analysis. β-actin served as the internal control.
