# Supplementary figures and images for: A novel hypoxic long noncoding RNA KB-1980E6.3 maintains breast cancer stem cell stemness via interacting with IGF2BP1 to facilitate c-Myc mRNA stability
Source: Oncogene. 2021 Jan 19;40(9):1609–27. doi: 10.1038/s41388-020-01638-9 (PMC7932928; doi:10.1038/s41388-020-01638-9)

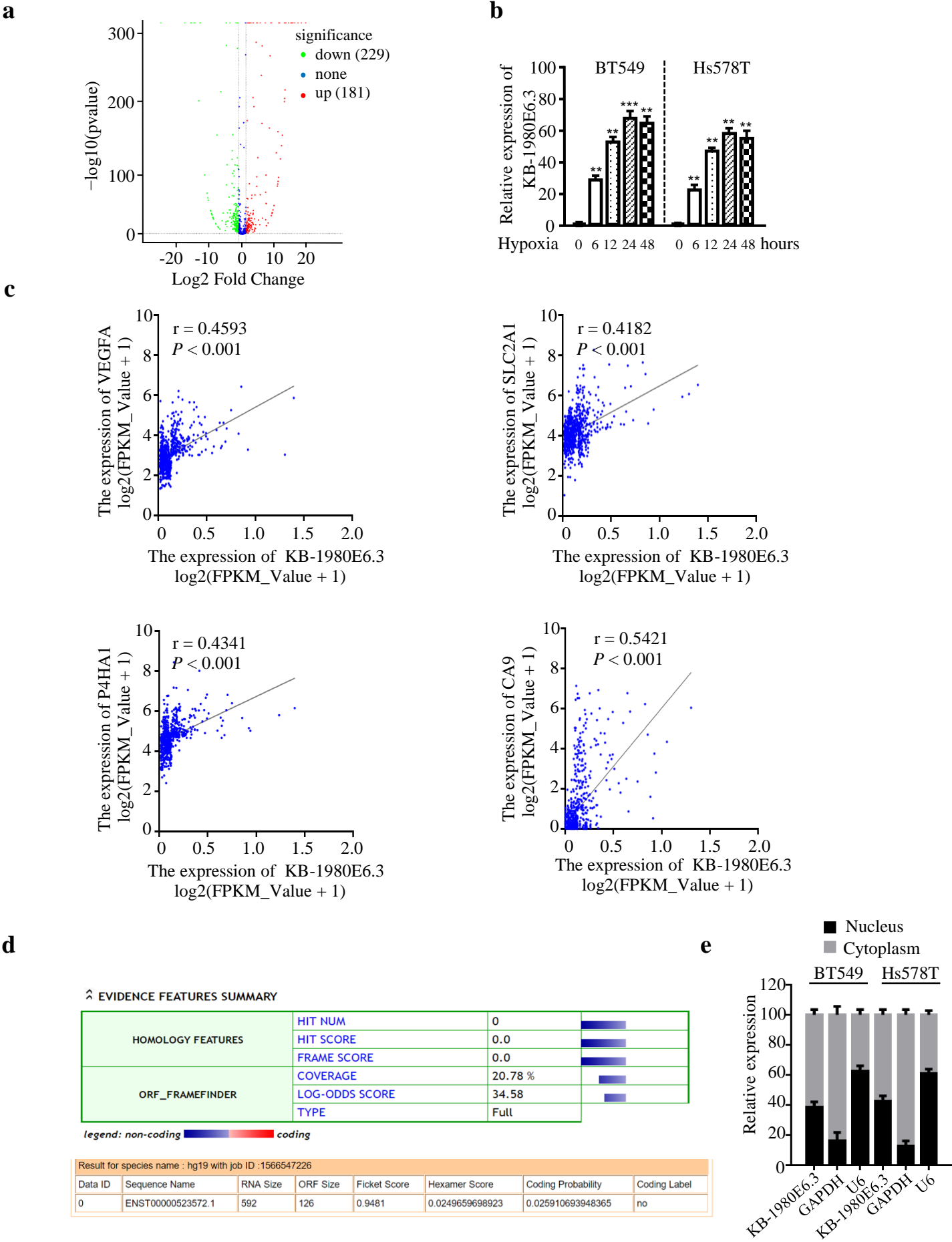

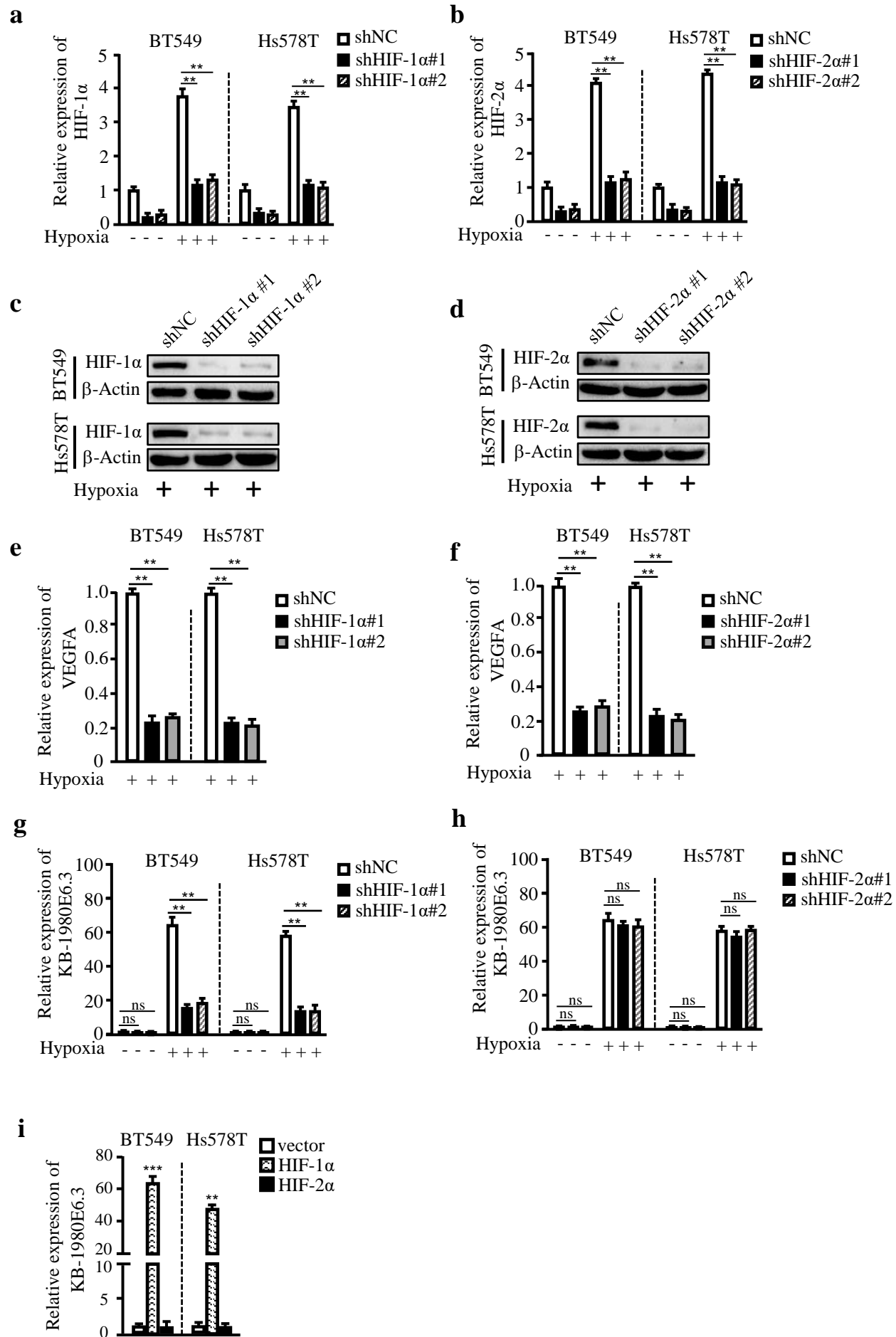

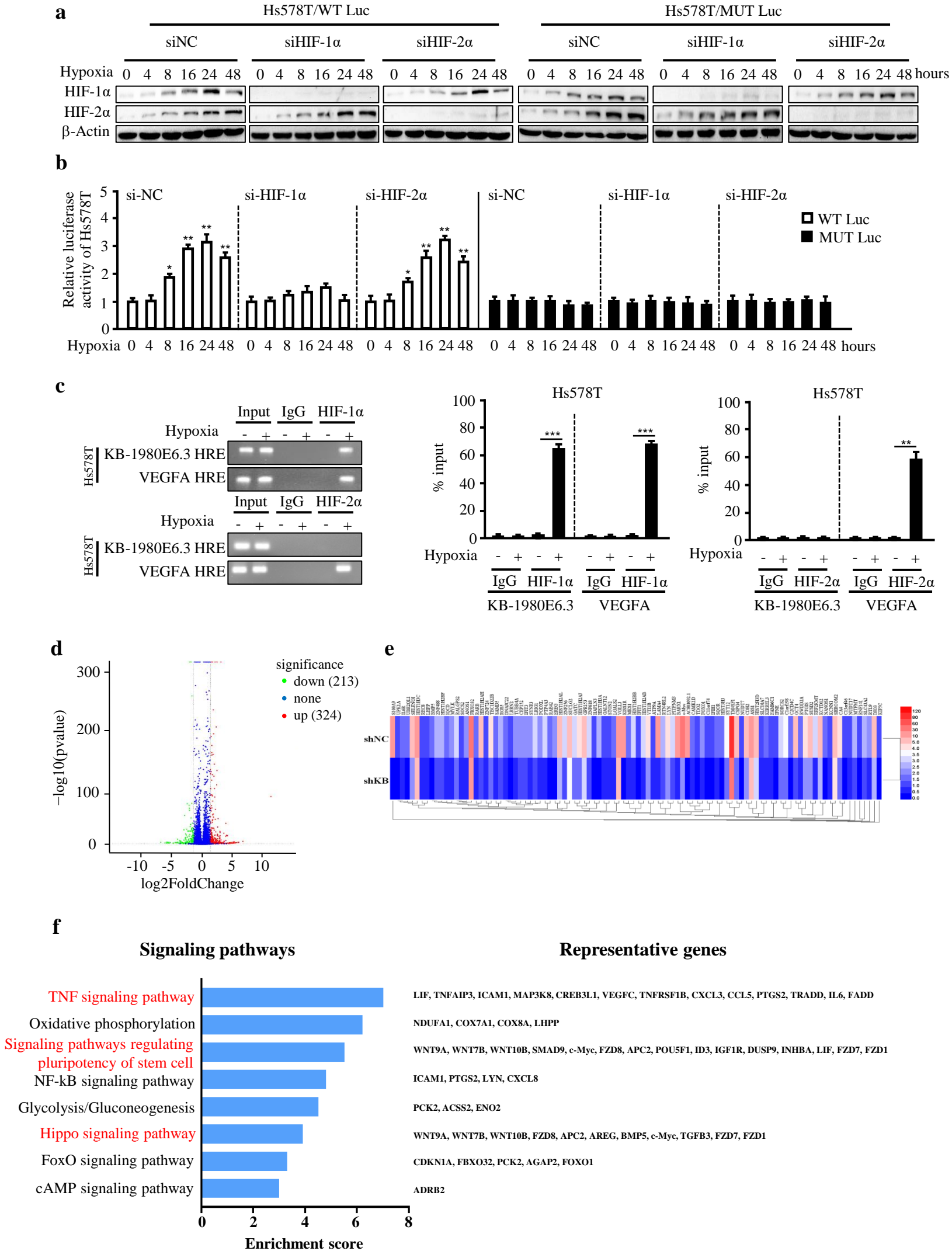

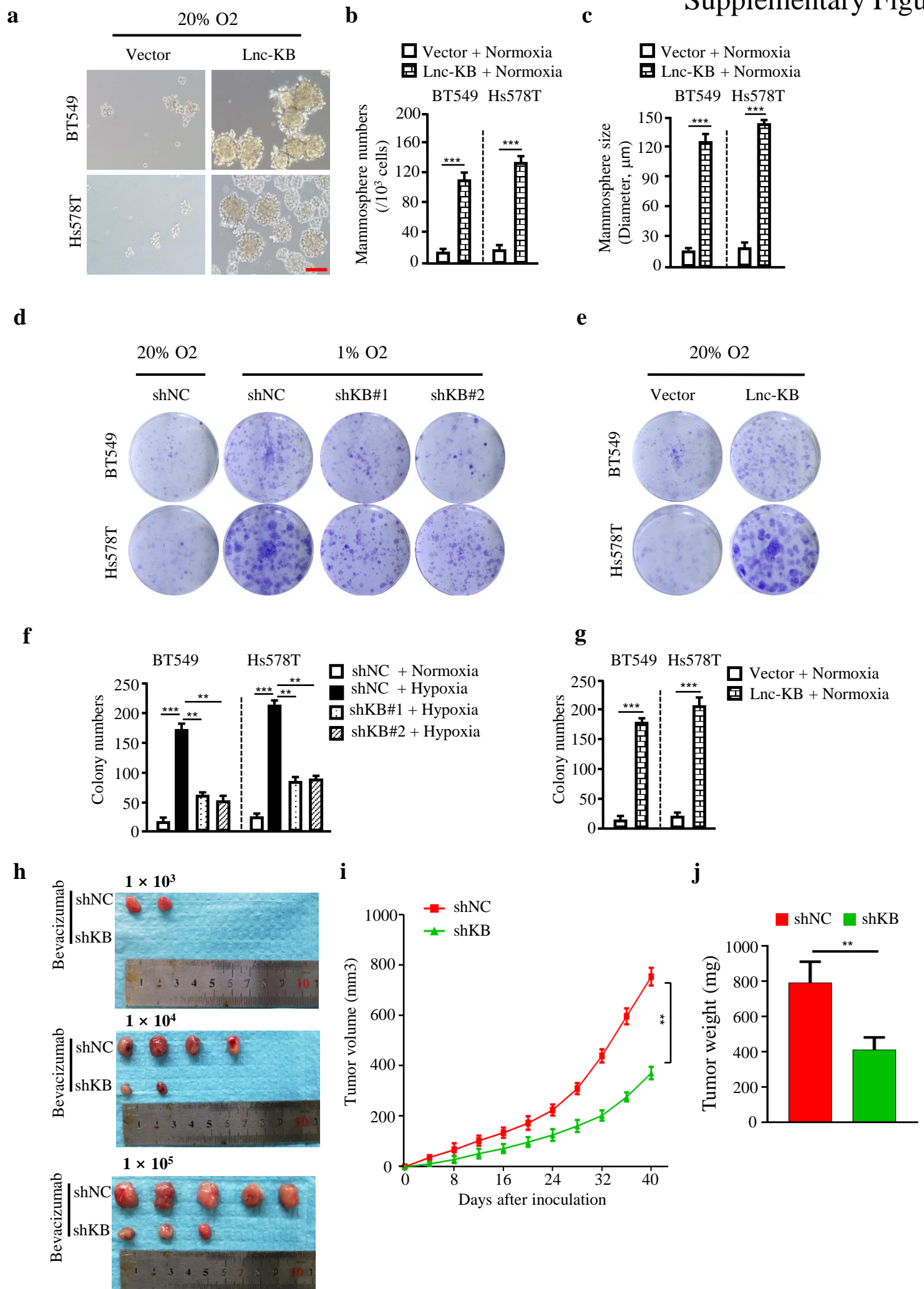

**a**

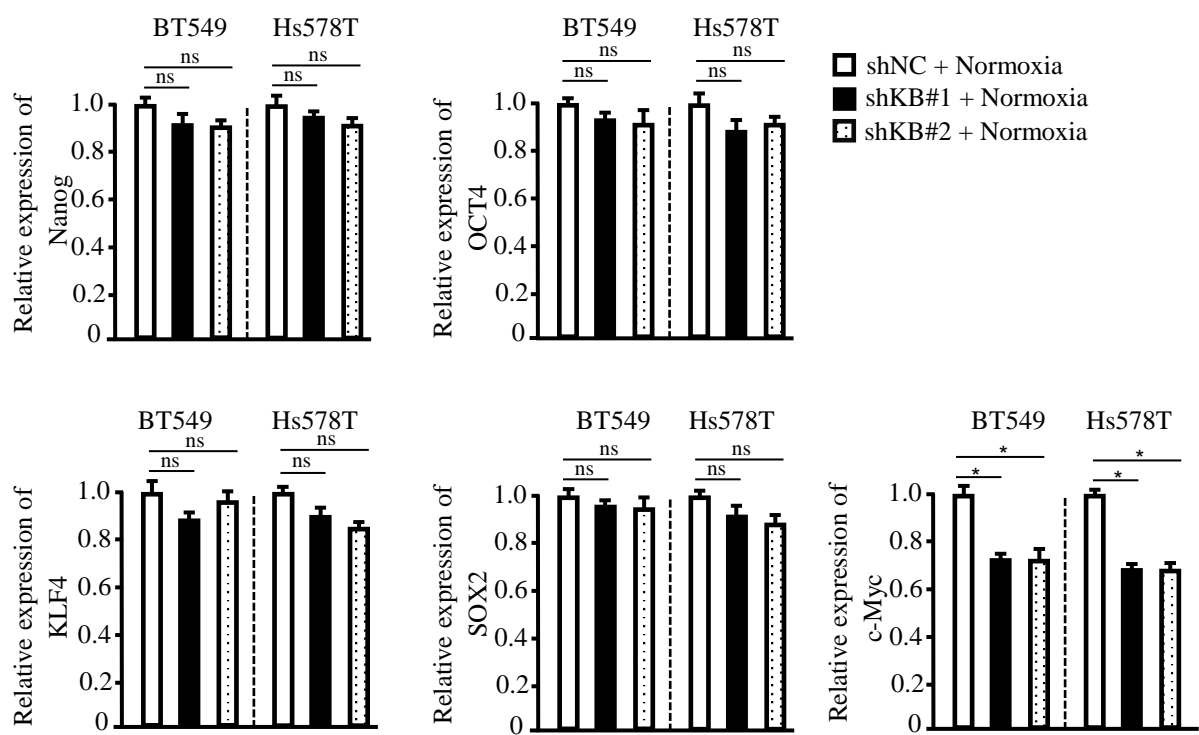

**b**

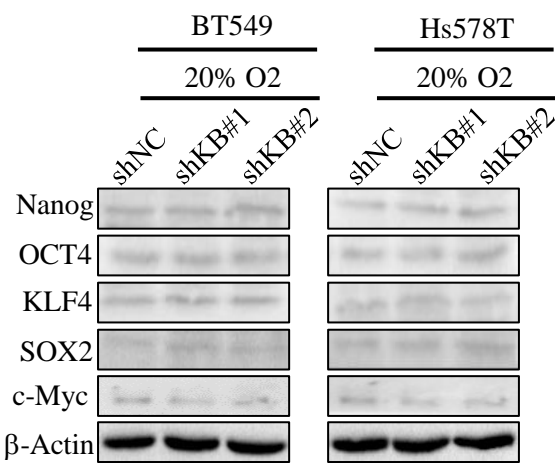

**a**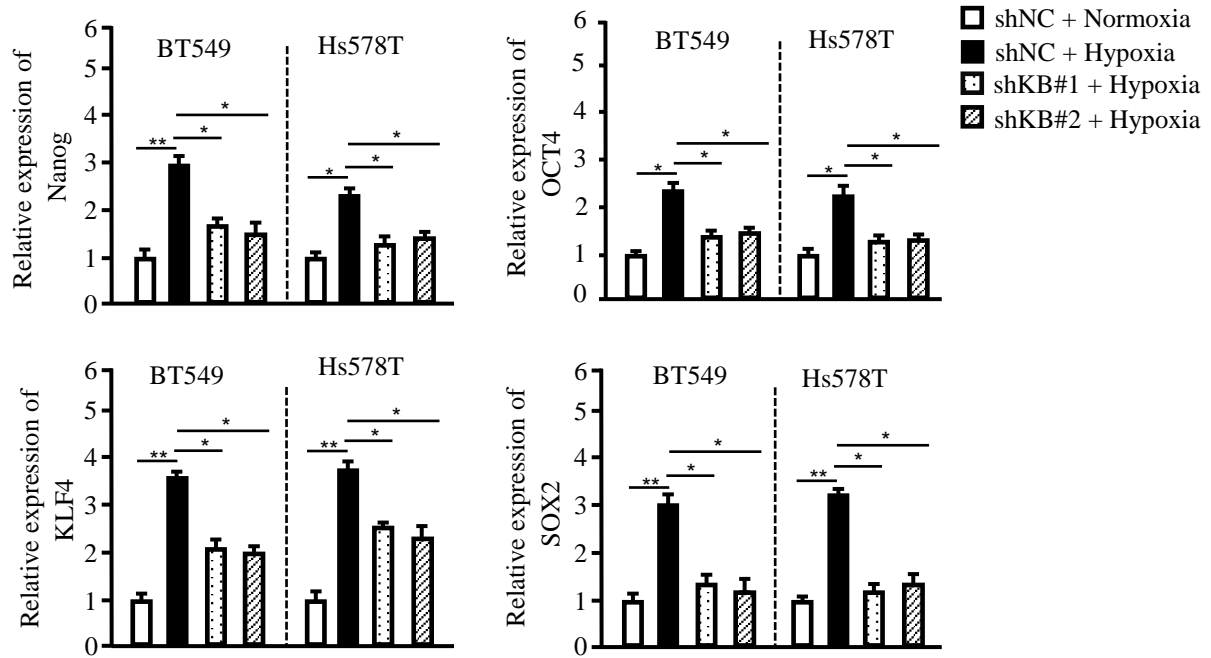**b**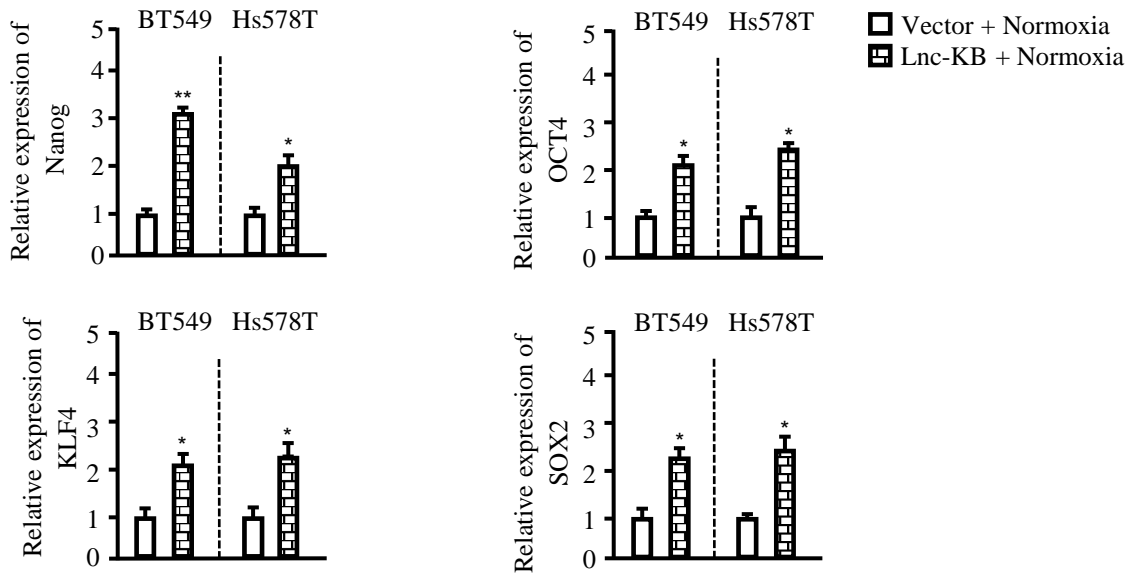**c**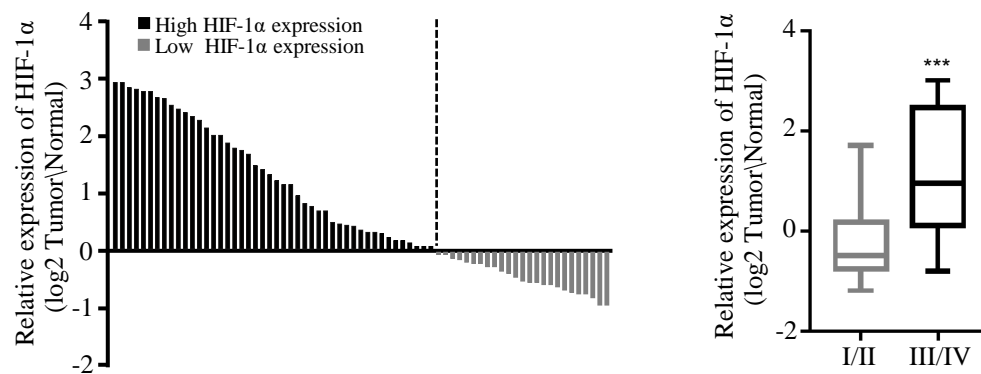**d**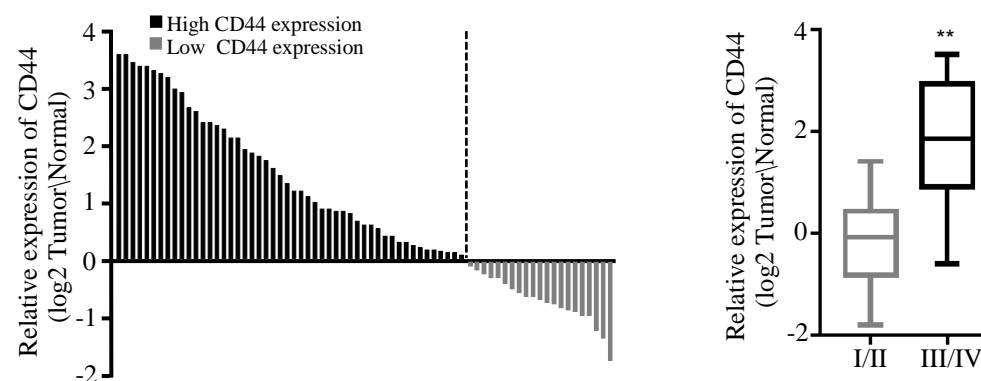

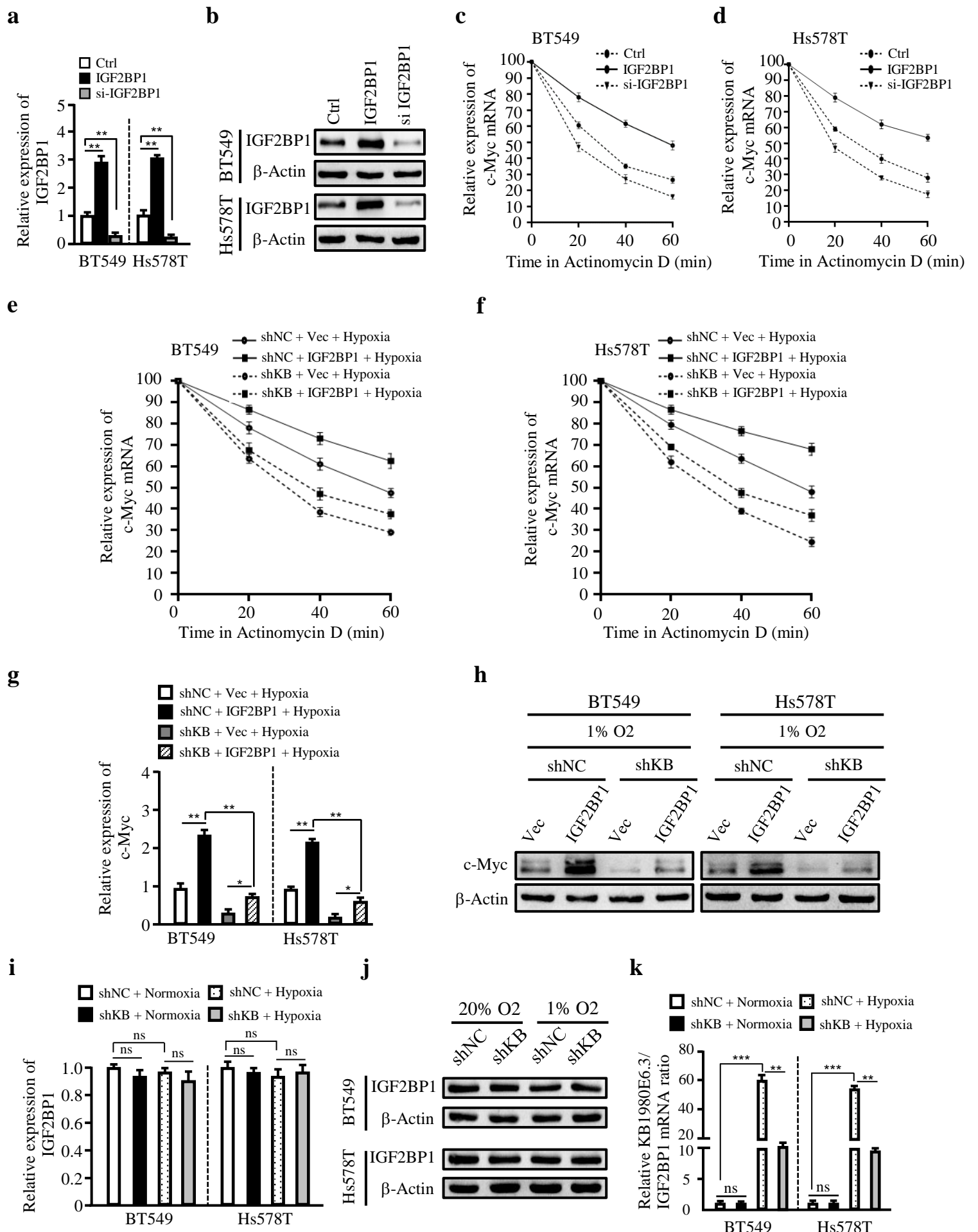

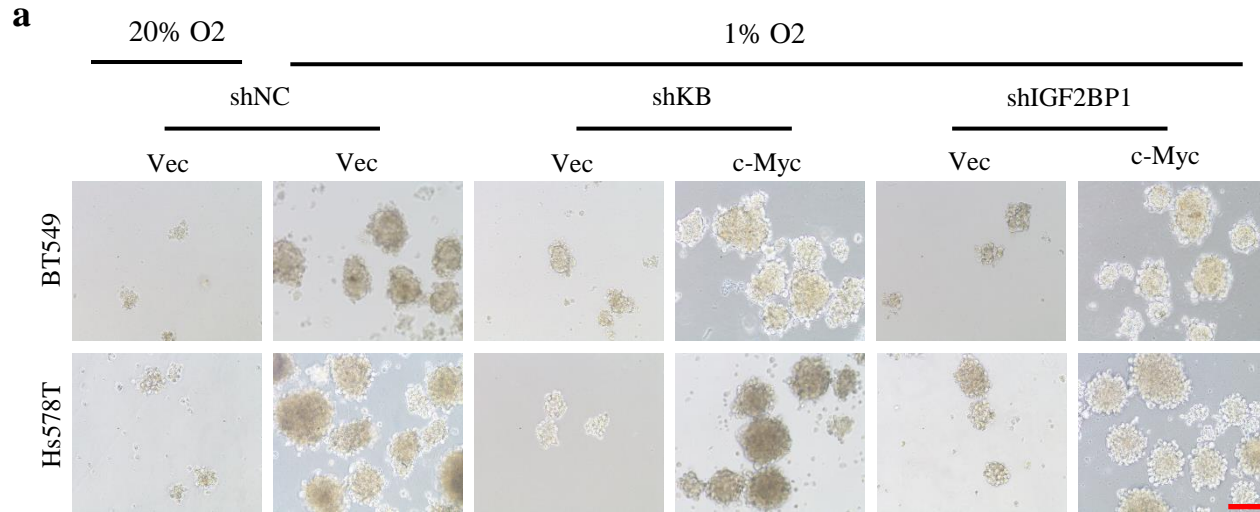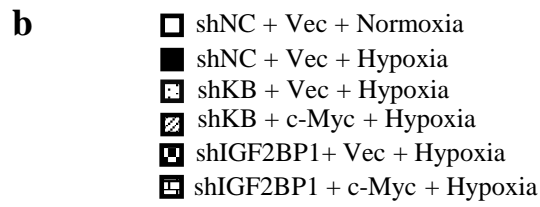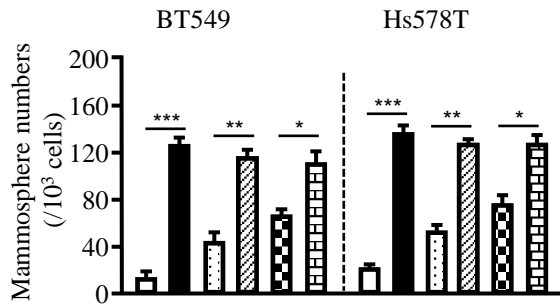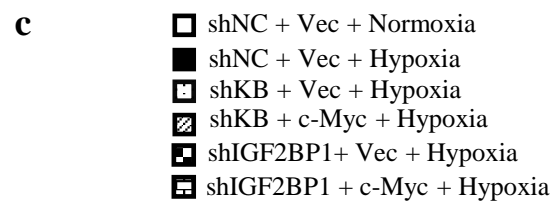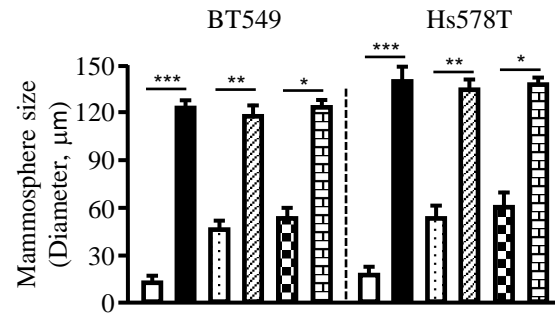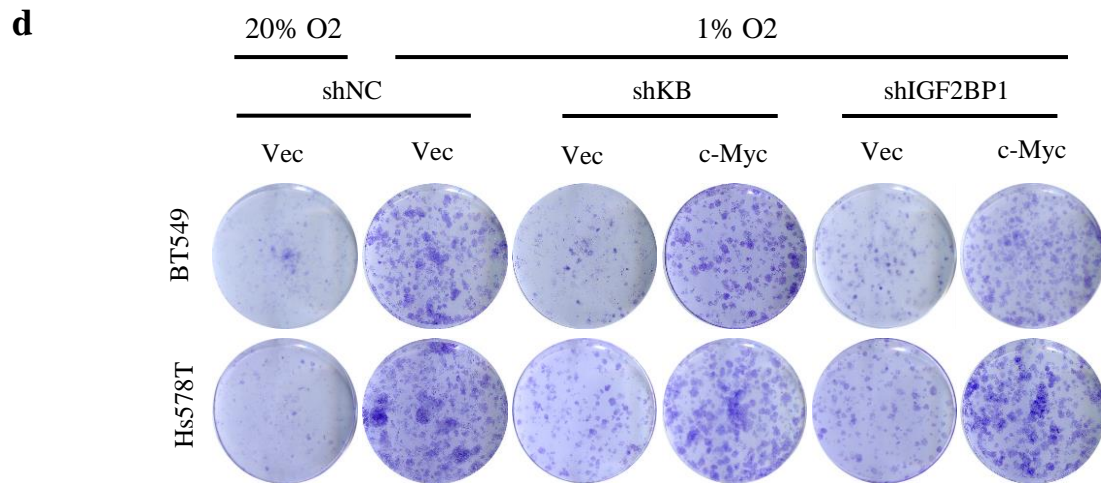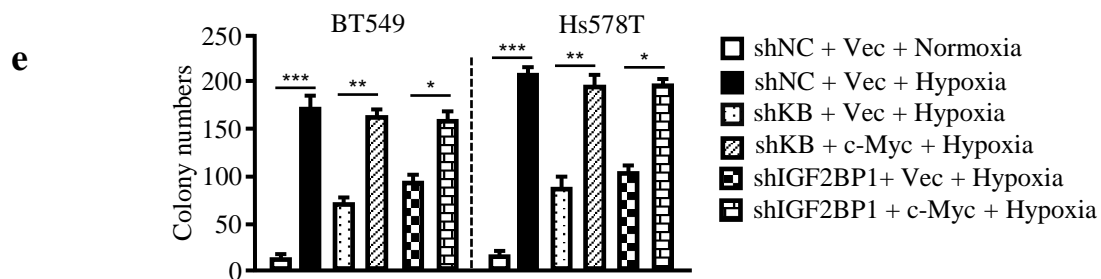

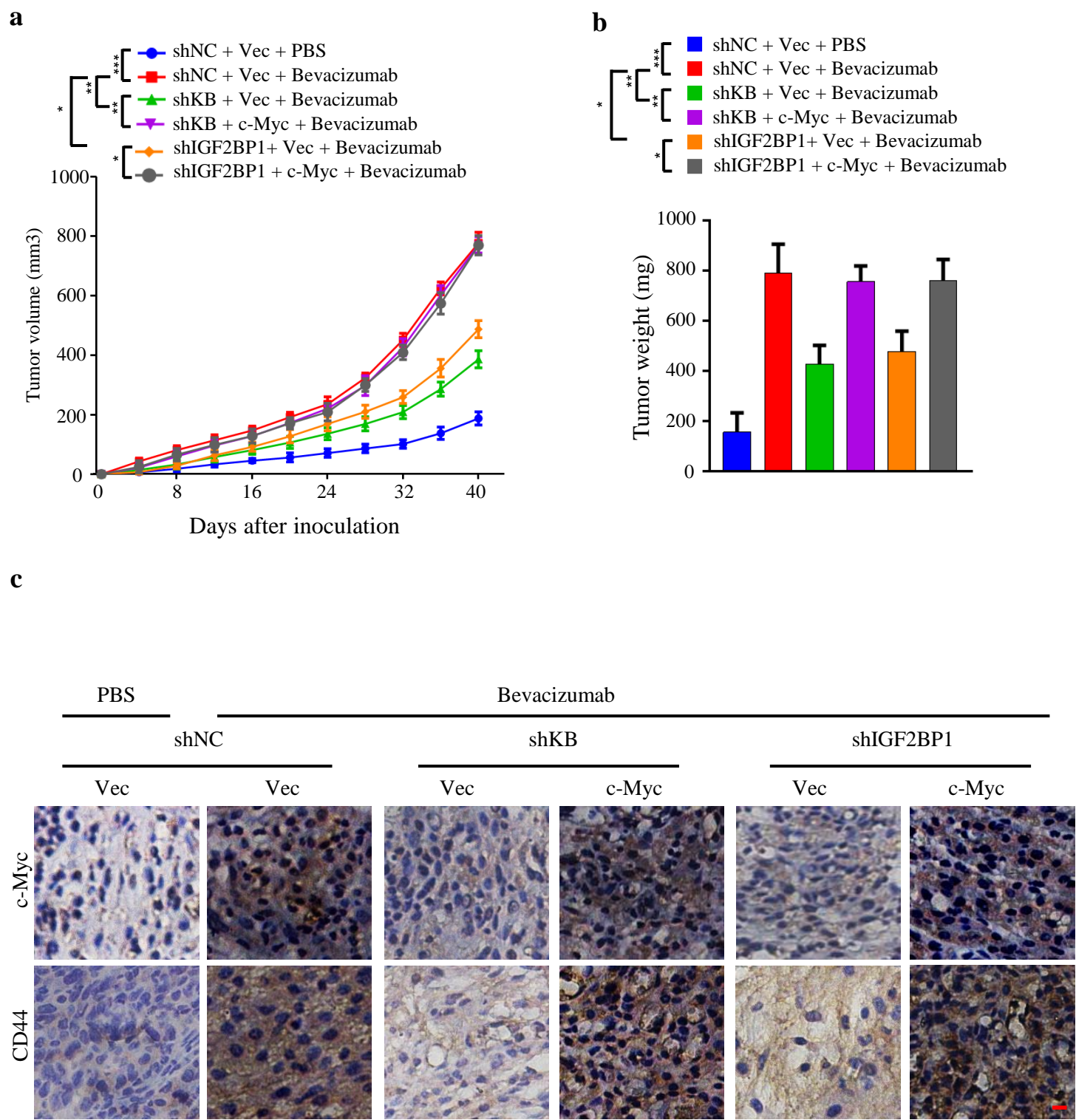

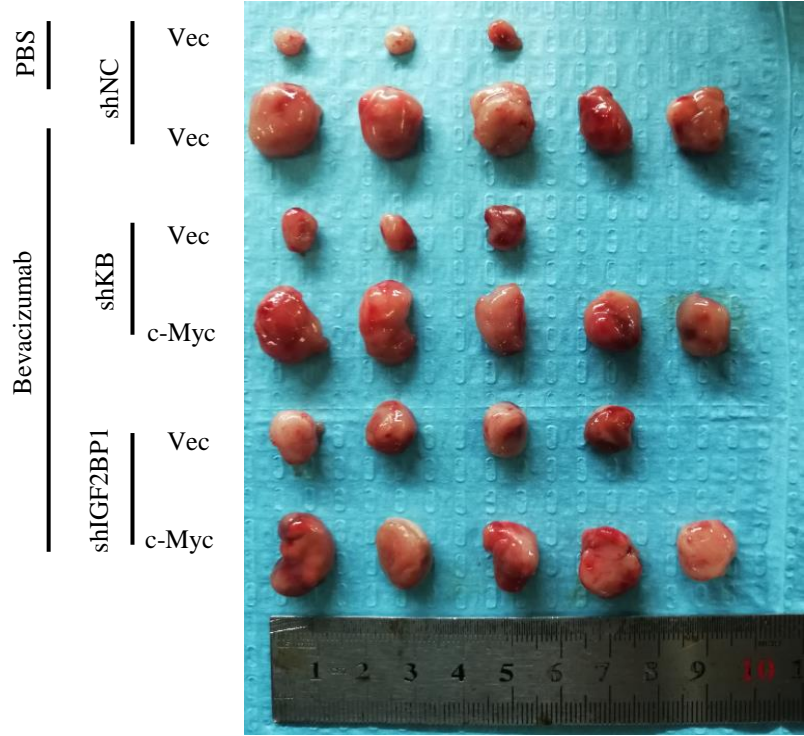

Supplement: Supplementary file 2 — Suppl Figures [file 41388_2020_1638_MOESM2_ESM.pdf]
