# Supplementary material for: A novel hypoxic long noncoding RNA KB-1980E6.3 maintains breast cancer stem cell stemness via interacting with IGF2BP1 to facilitate c-Myc mRNA stability
Source: Oncogene. 2021 Jan 19;40(9):1609–27. doi: 10.1038/s41388-020-01638-9 (PMC7932928; doi:10.1038/s41388-020-01638-9)
Supplement: Supplementary file 3 — Supplementary Figure legends [file 41388_2020_1638_MOESM3_ESM.docx]

**Supplementary Figure legends**

**Supplementary Figure 1. LncRNA KB-1980E6.3 is a hypoxic lncRNA. a** The volcano plot showing the differential lncRNAs in hypoxic BT549 cells compared with normoxic BT549 cells. **b** qRT-PCR was performed to determine lncRNA KB-1980E6.3 expression under hypoxia at designed time points. **c** The correlation between lncRNA KB-1980E6.3 and wellknown hypoxia-responsive genes in breast cancer tissues based on the data from TCGA database. **d** The noncoding nature of lncRNA KB-1980E6.3 was predicted by Coding Potential Calculator (CPC) and RNA coding potential assessment tool ([CPAT](https://sourceforge.net/projects/rna-cpat/)). **e** The localization of lncRNA KB-1980E6.3 in hypoxic BT549 and Hs578T cells was determined by subcellular fractionation assay. Data are shown as mean ± SD of three independent experiments (**, *P*<0.01; ***, *P*<0.001).

**Supplementary Figure 2. LncRNA KB-1980E6.3 is regulated by HIF-1α under hypoxia conditions. a, b** Knockdown efficiencies of HIF-1α and HIF-2α were confirmed at mRNA levels in BT549 and Hs578T cells under normoxia and hypoxia conditions. **c, d** Knockdown efficiencies of HIF-1α and HIF-2α were confirmed at protein levels in BT549 and Hs578T cells under hypoxia conditions. **e, f** qRT-PCR determined the VEGFA expression in HIF-1α or HIF-2α knockdown BT549 and Hs578T cells under hypoxia conditions. **g, h** qRT-PCR to determine the lncRNA KB-1980E6.3 expression in HIF-1α or HIF-2α knockdown BT549 and Hs578T cells under normoxia or hypoxia conditions. **i** BT549 and Hs578T cells were transfected with HIF-1α or HIF-2α encoding vector and cultured in normoxia conditions. The expression level of lncRNA KB-1980E6.3 was detected by qRT-PCR. Data are shown as mean ± SD of three independent experiments (ns: no significance; **, *P*<0.01; ***, *P*<0.001).

**Supplementary Figure 3. HIF-1α regulates lncRNA KB-1980E6.3 transcription through binding to the HRE region on its promoter. a** Hs578T cells were transfected with pGL3-lncRNA KB-1980E6.3 WT or HRE-mutant reporter combined with siHIF-1α or siHIF-2α under hypoxia conditions. The expression levels of HIF-1α or HIF-2α at the designed time point (0, 4, 8, 16, 24 and 48h) were examined by western blotting. **b** Hs578T cells were transfected with pGL3-lncRNA KB-1980E6.3 WT or HRE-mutant reporter, combined with siHIF-1α or siHIF-2α under hypoxia conditions. Dual-luciferase reporter activity was examined at designed hypoxia exposure times (0, 4, 8, 16, 24 and 48h). **c** ChIP assay was performed to determine the binding of HIF-1α or HIF-2α on the lncRNA KB-1980E6.3 promoter, the binding with VEGFA promoter served as a positive control for the HIF response in hypoxic Hs578T cells. **d** The volcano plot showing the differential genes between lncRNA KB-1980E6.3 knockdown and control cells under hypoxia conditions. **e** The heat map showing the top 100 downregulated genes between lncRNA KB-1980E6.3 knockdown and control cells under hypoxia conditions. **f** KEGG pathway analysis of differentially expressed genes in (**d**). Representative genes were shown in the right panel. Data are shown as mean ± SD of three independent experiments (*, *P*< 0.05; **, *P*<0.01; ***, *P*<0.001).

**Supplementary Figure 4. LncRNA KB-1980E6.3 plays a role in maintaining breast cancer stem cell properties.**

**a-c** Mammosphere formation abilities of lncRNA KB-1980E6.3 overexpressing BT549 and Hs578T cells were detected under normoxia conditions. The representative mammosphere, sphere numbers, and sphere size are shown in **(a), (b), and (c),** respectively. Scale bar, 100 μm. **d-g** Colony formations were measured using lncRNA KB-1980E6.3 knockdown or overexpressing tumor cells at indicated conditions. **h** Representative images of tumors in nude mice injected with 1×10^5^, 1×10^4^, or 1×10^3^ of sphere cells from Hs578T shNC or shKB. **i** Growth curve of tumors in nude mice injected with 1×10^5^ sphere cells from Hs578T shNC or shKB. **j** Weight of mice tumors in (**i**) on the day of sacrifice. Data are shown as mean ± SD of three independent experiments (**, *P*< 0.01; ***, *P*<0.001).

**Supplementary Figure 5. LncRNA KB-1980E6.3 has little impact on the expression of pluripotency-associated markers under normoxia conditions. a** The mRNA levels of c-Myc, KLF4, SOX2, OCT4, and Nanog were assessed by qRT-PCR in lncRNA KB-1980E6.3 knockdown BT549 and Hs578T cells under normoxia conditions. **b** The protein levels of c-Myc, KLF4, SOX2, OCT4, and Nanog were evaluated by western blotting in lncRNA KB-1980E6.3 knockdown BT549 and Hs578T cells under normoxia conditions. Data are shown as mean ± SD of three independent experiments (ns: no significance; *, *P*< 0.05).

**Supplementary Figure 6. LncRNA KB-1980E6.3 regulates expression of pluripotency-associated markers under hypoxia conditions. a** KLF4, SOX2, OCT4 and Nanog expressions were assessed by qRT-PCR in lncRNA KB-1980E6.3 knockdown breast cancer cells under hypoxia. **b** qRT-PCR was used to determine KLF4, SOX2, OCT4 and Nanog mRNA levels in the lncRNA KB-1980E6.3 overexpressing tumor cells under normoxia. **c** The heights of the columns in the chart represent the log2-transformed fold changes (tumor vs normal) in HIF-1α expression in 71 paired breast cancer tissues and adjacent non-cancerous tissues (left panel). The relative log2-transformed fold changes (tumor vs normal) of HIF-1α in 71 cases of breast tumors with different clinical stages was measured by qRT-PCR (right panel). **d** The heights of the columns in the chart represent the log2-transformed fold changes (tumor vs normal) in CD44 expression in 71 paired breast cancer tissues and adjacent non-cancerous tissues (left panel). The relative log2-transformed fold changes (tumor vs normal) of CD44 in 71 cases of breast tumors with different clinical stages was measured by qRT-PCR (right panel). Data are shown as mean ± SD of three independent experiments (*, *P*< 0.05; **, *P*< 0.01; ***, *P*<0.001).

**Supplementary Figure 7. LncRNA KB-1980E6.3 recruits IGF2BP1 to enhance c-Myc mRNA stability in breast cancer cells. a, b** IGF2BP1 mRNA and protein levels were determined in normoxic BT549 and Hs578T cells transfected with IGF2BP1 encoding vector or siIGF2BP1 by qRT-PCR and western blotting. **c, d** The BT549 and Hs578T cells described in (**a, b**) were treated with Actinomycin D for the indicated times, and the c-Myc mRNA levels were measured by qRT-PCR. **e, f** LncRNA KB-1980E6.3 knockdown BT549 and Hs578T cells transfected with or without IGF2BP1 coding vector and cultured under hypoxia conditions. The cells were treated with Actinomycin D for the indicated times, and c-Myc mRNA levels were measured by qRT-PCR. **g, h** c-Myc mRNA and protein levels were examined in the engineered cells in **(e, f)**. **i, j** IGF2BP1 mRNA and protein levels were detected in the lncRNA KB-1980E6.3-knocked down and the control cells under normoxia and hypoxia conditions. **k** The lncRNA KB-1980E6.3/IGF2BP1 ratio in the lncRNA KB-1980E6.3 knockdown and the control cells under normoxia and hypoxia conditions. Data are shown as mean ± SD of three independent experiments (ns: no significance; *, *P*< 0.05; **, *P*< 0.01; ***, *P*<0.001).

**Supplementary Figure 8. Ectopic c-Myc partially rescues BCSCs formation ability repressed by lncRNA KB-1980E6.3 or IGF2BP1 gene loss in hypoxia. a-c** LncRNA KB-1980E6.3 or IGF2BP1 knockdown BT549 and Hs578T cells were restored with or without c-Myc, and mammosphere formation was determined. Representative images of spheres in each group of engineered tumor cells, mammosphere numbers, and mammosphere size are shown in **(a), (b), and (c),** respectively. Scale bar, 100 μm. **d, e** The engineered breast cancer cells were seeded in 6‐well plates, and cell colony formation was measured. Representative images of colony **(d)** and colony numbers **(e)** are shown. Data are shown as mean ± SD of three independent experiments (*, *P*< 0.05; **, *P*< 0.01; ***, *P*<0.001).

**Supplementary Figure 9. LncRNA KB-1980E6.3-dependent c-Myc stability is essential for Hs578T-derived breast tumorigenesis in vivo.** 1×10^5^ sphere cells derived from engineered Hs578T including Hs578T/shKB/vector, Hs578T/shKB/c-Myc, Hs578T/sh IGF2BP1/vector, Hs578T/sh IGF2BP1/c-Myc, and Hs578T/shNC/vector control cells were injected into nude mice, and mice were administrated with bevacizumab at 10 mg/kg or vehicle (PBS). **a** Growth curve of tumors in nude mice. **b** Tumor weight in **(a)** on the day of sacrifice. **c** Representative IHC staining of c-Myc and CD44 protein in mouse xenograft tumor tissues, scale bar, 50 μm. Data are shown as mean ± SD of three independent experiments (*, *P*< 0.05; **, *P*< 0.01; ***, *P*<0.001).

**Supplementary Figure 10. LncRNA KB-1980E6.3-dependent c-Myc stability is essential for BT549-derived breast tumorigenesis in vivo.** 1×10^5^ sphere cells derived from the engineered BT549 including BT549/shKB/vector, BT549/shKB/c-Myc, BT549/shIGF2BP1/vector, BT549/shIGF2BP1/c-Myc, and BT549/shNC/vector control cells were injected into nude mice, and the mice were administrated with bevacizumab at 10 mg/kg or vehicle (PBS). The representative images of tumors in each mouse group.
