# Supplementary material for: A novel hypoxic long noncoding RNA KB-1980E6.3 maintains breast cancer stem cell stemness via interacting with IGF2BP1 to facilitate c-Myc mRNA stability
Source: Oncogene. 2021 Jan 19;40(9):1609–27. doi: 10.1038/s41388-020-01638-9 (PMC7932928; doi:10.1038/s41388-020-01638-9)
Supplement: Supplementary file 4 — Supplementary Table 1 [file 41388_2020_1638_MOESM4_ESM.docx]

**Supplementary Table 1.** **The sequences of lncRNAKB-1980E6.3**

>ENST00000523572.1 (AP002852.1) length=592

GTGCGCTGTGTTCTACGTGCTCTGTGCTGTGCGCTGTGCTGTGTGCTGAGCTGTGCTGGC

ATCGTTCTCACTCCTCCTGGTGGCTGTGGGAGGATGGACTGGTGGTTTTGGAGGAATCCA

GAGAAATGGCTGAATCCCACCTCTGAGCTGGAGCAGAGAAGGGAAAAAAGAAGTCTGGTT

GGTCTGAGAATGAACCCCACAGTCCAGAGGCAGCAGTGAGCTCCTGGGAATCTGGATAAA

CTTGAGGAGGACACTGTTCATTGTACTTTACGCCAACGTGGACTGGAGGATACAACCCGT

TTTATTTCAGTCCCAGAGTGACCTCGGATGACACGTGGGAGAGCGCTGTACATTCATGCA

TATATATACATGTATGTGATTTGTAACATGTATATTATGTGGTTTATATAACAGATGCAT

GACATAAACATGTACCGGTAGAGACGTAGAGAATTCTTTATAAAAAGTGACATAATATAA

TGAATCAATAAATCTATATATATAGTATACATTGTATATTATGTAAATGTAACATGGCAT

ATTATAAAACATCTATAAAACAAACATAACACATATACTGTTTTATATATGA
