# Supplementary material for: A novel hypoxic long noncoding RNA KB-1980E6.3 maintains breast cancer stem cell stemness via interacting with IGF2BP1 to facilitate c-Myc mRNA stability
Source: Oncogene. 2021 Jan 19;40(9):1609–27. doi: 10.1038/s41388-020-01638-9 (PMC7932928; doi:10.1038/s41388-020-01638-9)
Supplement: Supplementary file 5 — Supplementary Table 2 [file 41388_2020_1638_MOESM5_ESM.docx]

| **Supplementary Table 2. Sequences of siRNAs used in this study** | | |
| --- | --- | --- |
| Gene name | sense | antisense |
| si- HIF-1α | 5’-CCGCUGGAGACACAAUCAUAU-3’ | 5’-AUAUGAUUGUGUCUCCAGCGG-3’ |
| si- HIF-2α | 5’-GCGCAAAUGUACCCAAUGAUA-3’ | 5’-UAUCAUUGGGUACAUUUGCGC-3’ |
| si-IGF2BP1 | 5’-CCUGGCCCAUAAUAACUUUTT-3’ | 5’-AAAGUUAUUAUGGGCCAGGTT-3’ |
| si-KB-1980E6.3-1 | 5’-CUGGGAAUCUGGAUAAACUTT-3’ | 5’-AGUUUAUCCAGAUUCCCAGTT-3’ |
| si-KB-1980E6.3-2 | 5’-GGAGAGCGCUGUACAUUCATT-3’ | 5’-UGAAUGUACAGCGCUCUCCTT-3’ |
| si-c-Myc | 5’-GUGCAGCCGUAUUUCUACUTT-3’ | 5’-AGUAGAAAUACGGCUGCACTT-3’ |
|  |  |  |
|  |  |  |
