# Supplementary material for: A novel hypoxic long noncoding RNA KB-1980E6.3 maintains breast cancer stem cell stemness via interacting with IGF2BP1 to facilitate c-Myc mRNA stability
Source: Oncogene. 2021 Jan 19;40(9):1609–27. doi: 10.1038/s41388-020-01638-9 (PMC7932928; doi:10.1038/s41388-020-01638-9)
Supplement: Supplementary file 6 — Supplementary Table 3 [file 41388_2020_1638_MOESM6_ESM.docx]

| **Supplementary Table 3. The shRNA sequences specifically against the target gene** | |
| --- | --- |
| Gene name | Sequences |
| shHIF1α-1 | 5’-CCGCUGGAGACACAAUCAUAU-3’ |
| shHIF1α-2 | 5’-CCAUAUAGAGAUACUCAAATT-3’ |
| shHIF2α-1 | 5’-GCGCAAAUGUACCCAAUGAUA-3’ |
| shHIF2α-2 | 5’-CAAUAGCCCUGAAGACUAUTT-3’ |
| shKB-1980E6.3-1 | 5’-CUGGGAAUCUGGAUAAACUTT-3’ |
| shKB-1980E6.3-2 | 5’-GGAGAGCGCUGUACAUUCATT-3’ |
| shIGF2BP1 | 5’-CCUGGCCCAUAAUAACUUUTT-3’ |
