# Supplementary material for: A novel hypoxic long noncoding RNA KB-1980E6.3 maintains breast cancer stem cell stemness via interacting with IGF2BP1 to facilitate c-Myc mRNA stability
Source: Oncogene. 2021 Jan 19;40(9):1609–27. doi: 10.1038/s41388-020-01638-9 (PMC7932928; doi:10.1038/s41388-020-01638-9)
Supplement: Supplementary file 7 — Supplementary Table 4 [file 41388_2020_1638_MOESM7_ESM.docx]

| \| **Supplementary Table 4. Primers used for qRT-PCR and ChIP analysis.** \| \| \| \| --- \| --- \| --- \| \| Gene name \| Forward \| Reverse \| \| β-actin \| CCACTGGCATCGTGATGGA \| CGCTCGGTGAGGATCTTCAT \| \| HIF1α \| TCTAGACTCGAGTACAAGGCAGCAGAAAC \| TCTAGAGTTTGTGCAGTATTGTAGCC \| \| HIF2α \| TGGGATCTAACAGGAACAGC \| CTAAATAGCCAGACAAGGGT \| \| VEGFA \| ATGAACTTTCTGCTCTCTGG \| TCATCTCTCCTATGTGCTGGC \| \| KB-1980E6.3 \| GTTCATTGTACTTTACGCCAA \| ATATATGCATGAATGTACAGC \| \| KB-1980E6.3 HRE \| TCTGTGCTGTGTTGCATGCT \| CTTACATAAACAAAGCACAC \| \| c-Myc \| GGGCTTTATCTAACTCGCTGTA \| GCTATGGGCAAAGTTTCGTG \| \| SOX2 \| ATGACCAGCTCGCAGACCTAC \| TTGACCACCGAACCCATGGAG \| \| KLF4 \| GAACTGACCAGGCACTACCG \| TTCTGGCAGTGTGGGTCATA \| \| OCT4 \| TGGGCTCGAGAAGGATGTG \| GCATAGTCGCTGCTTGATCG \| \| Nanog \| TCCAGCAGATGCAAGAACTCTCCA \| CACACCATTGCTATTCTTCGGCCA \| \| CD44 \| CTCTCGGACGGAGGCCGCTGACC \| AGAAGGGCACGTGGTGATTCCCCG \| \| GAPDH \| GAAGGTGAAGGTCGGAGTC \| AAGATGGTGATGGGATTTC \| \| c-Myc CRD \| AACACACAACGTCTTGGAG \| TTACGCACAAGAGTTCCGTAG \| \| IGF2BP1 \| CAGGAGATGGTGCAGGTGTTTATCC \| GTTTGCCATAGATTCTTCCCTGAGC \| \| **Sequences of primers used for pcDNA3.3-IGF2BP1 construction** \| \| \| \| 1-1731 \| F:ATGAACAAGCTTTACATCGG \| R:CTTCCTCCGTGCCTGGGCCTG \| \| 1-582 \| F:ATGAACAAGCTTTACATCGG \| R:CACTTGCTGCTGCTTGGCTG \| \| 583-1170 \| F:GACATCCCCCTTCGGCTCCT \| R:AACGCTGCTGGGAGGCGGCG \| \| 1171-1731 \| F:ACTGGGGCTGCTCCCTATAG \| R:CTTCCTCCGTGCCTGGGCCTG \| \| **Sequences of primers used for pcDNA3.3-KB-1980E6.3 construction** \| \| \| \| 1-200 \| F:GTGCGCTGTGTTCTACGTGC \| R:GTGGGGTTCATTCTCAGACC \| \| 201-400 \| F:AGTCCAGAGGCAGCAGTGAG \| R:ACATAATATACATGTTACAA \| \| 401-592 \| F:GGTTTATATAACAGATGCAT \| R:TCATATATAAAACAGTATAT \| \| **Primers for in vitro transcription The T7 RNA polymerase sequence(T7) was 5'taatacgactcactataggg’3** \| \| \| \| KB-1980E6.3 (sense） \| F: (T7)GTGCGCTGTGTTCTACGTGCTCTGTGCT \| R:TCATATATAAAACAGTATATGTGTTATG \| \| KB-1980E6.3 (antisense） \| F:(T7)TCATATATAAAACAGTATATGTGTTATG \| R:GTGCGCTGTGTTCTACGTGCTCTGTGCT \| \| **Primers for antisense oligomer affinity pull-down assays** \| \| \| \| KB-1980E6.3 sense oligo DNA \| 5' (biotin-)ACGTTAGACACAGCCTCCGTG’3 \| \| \| KB-1980E6.3 antisense oligo DNA \| 5' (biotin-)AAGAAUUCUCUACGUCUCUACC’3 \| \| |  |  |  |
| --- | --- | --- | --- | --- | --- | --- | --- | --- | --- | --- | --- | --- | --- | --- | --- | --- | --- | --- | --- | --- | --- | --- | --- | --- | --- | --- | --- | --- | --- | --- | --- | --- | --- | --- | --- | --- | --- | --- | --- | --- | --- | --- | --- | --- | --- | --- | --- | --- | --- | --- | --- | --- | --- | --- | --- | --- | --- | --- | --- | --- | --- | --- | --- | --- | --- | --- | --- | --- | --- | --- | --- | --- | --- | --- | --- | --- | --- | --- | --- | --- | --- | --- | --- | --- | --- | --- | --- | --- | --- | --- | --- | --- | --- | --- | --- | --- | --- | --- | --- |
